# Place field dynamics in retrosplenial cortex compared to hippocampus

**DOI:** 10.1016/j.pneurobio.2025.102867

**Published:** 2025-12-10

**Authors:** Zaneta Navratilova, Dhruba Banerjee, Fjolla Muqolli, Jordan Zhang, Sunil P. Gandhi, Bruce L. McNaughton

**Affiliations:** a University of California-Irvine, 2205 McGaugh Hall, Irvine, CA 92697, USA; b University of Lethbridge, 4401 University Drive, Lethbridge, AB T1K 3M4, Canada

**Keywords:** Hippocampal-cortical interactions, Place cells, CA1, Global remapping, Memory index theory

## Abstract

The encoding, storage, and updating of memories in cortical networks are poorly understood. In retrosplenial cortex (RSC), cells respond to the animal’s position as it traverses a real or virtual (VR) linear track. Most position correlated cells (PCCs) in RSC require an intact hippocampus to form, but survive subsequent hippocampal damage. To examine whether RSC and hippocampal PCCs undergo remapping and spatial tuning development in parallel or sequentially, neuronal activity in RSC or CA1 was recorded using two-photon calcium imaging in mice running on VR tracks. RSC PCC activity underwent global remapping like CA1, with approximately the same dynamics of tuning development in the novel context. However, fields in RSC did not show place field expansion, in familiar or novel environments. Thus, while most properties of global remapping are shared between RSC and CA1, place field shift and expansion are notably restricted to hippocampus. Thus, our data suggests that RSC place specificity is either not ‘inherited’ directly from hippocampus or the hippocampal influence on RSC PCC formation may be restricted to hippocampal spikes occurring in the early phase of the theta rhythm (and thus late within the place field).

The retrosplenial cortex (RSC) has been implicated in memory processing through loss-of-function, anatomical, and functional imaging studies. Patients with brain damage localized to RSC experience anterograde and retrograde amnesia (Gainotti et al., 1998; Osawa et al., 2007; Valenstein et al., 1987), while excitotoxic lesions or inactivation of the region in experimental animals disrupt learning in spatial navigation tasks and recall of associations (Harker and Whishaw, 2002; Sutherland et al., 1988; Vann et al., 2009; Todd et al., 2016). Anatomical tracing studies across species show that RSC selectively receives inputs from other regions associated with memory, including the hippocampus and entorhinal cortex (Cenquizca and Swanson, 2007; Miyazaki et al., 2012) as well as strong inputs from visual cortex (Vélez-Fort et al., 2014; Vogt and Miller, 1983; van Groen and Wyss, 1992). Additionally, RSC becomes functionally engaged during memory tasks in humans (Wolbers and Büchel, (2005). Similarities between effects of RSC and hippocampal lesions suggest that RSC is a hub of distribution of hippocampal output to cortex, consistent with anatomical (van Groen and Wyss, 1992) and voltage imaging data (Abadchi et al., 2020).

At the cellular level, activity patterns in RSC show notable similarities to those in other memory-associated brain regions. The most striking, and first discovered, pattern of activity in the temporal lobe is the highly localized firing of “place cells” in the hippocampus (O’Keefe and Dostrovsky, 1971). In recent years, recordings have been made from RSC neurons in live animals navigating through real or virtual reality (VR) environments. In mice running through a linear corridor, position-correlated cells (PCCs) were found within the RSC (Fischer et al., 2020; Mao et al., 2017; 2018). Each PCC fires when the animal enters one or a few specific portions of the track, known as that cell’s place fields, such that when the mouse runs the length of the corridor, the corresponding PCCs become sequentially active and cover the space relatively uniformly. Interestingly, PCCs in RSC and place cells in hippocampal CA1 both demonstrate spatial context specificity (Miller et al., 2021; Subramanian et al., 2024) and are driven by similar signals, including visual cues, distance traveled, rewards, and trajectories (Mao et al., 2017; Vedder et al., 2017; Miller et al., 2019; Powell et al., 2020; Mao et al., 2020; Demchuk et al., 2024). In fact, PCCs in RSC depend on an intact hippocampus to form (Mao et al., 2018; Esteves et al., 2020), but once established these sequences survive hippocampal lesions (Esteves et al., 2023). These findings raise an intriguing possibility that PCCs in RSC support memory processing, in a manner similar to how place cells function in hippocampus. But whether hippocampus directly imposes spatial selectivity on cortex or merely enables some other intrinsic dynamics remains unclear and the present study was designed to elucidate this issue by comparing hippocampal and RSC dynamics of spatial tuning development in novel environments.

Hippocampal place cell representations change, or remap (Hill, 1978; Muller and Kubie, 1987; Sanders et al., 2020; Plitt and Giocomo 2021; Keinath et al., 2022) in response to new experiences, displaying properties expected of a network specialized for memory processing. Major alterations to the environment can induce global remapping, such that hippocampal activity in one spatial context does not predict activity in another (Kubie and Muller, 1991; Leutgeb et al., 2005). A sparse subset of cells is allocated to represent each location within each context, with overlap close to chance levels, increasing storage capacity while avoiding interference (Marr, 1971; McNaughton and Morris, 1987; Treves and Rolls, 1991). Within minutes of exposure to a new context, a distinct representation emerges, with many fields forming on the very first exposure (Hill, 1978; Wilson and McNaughton, 1993; Frank et al., 2004; Navratilova et al., 2012; Dong et al., 2021). Detailed lap-by-lap analysis of field formation has yielded insights that link plasticity mechanisms at the molecular level to learning and memory more broadly. In particular, repeated traversal of the same sequence of locations within the same session causes the size of CA1 and CA3 place fields to increase, and their center of mass (COM) to shift in the opposite direction of motion (Mehta et al., 1997; 2000), a process dependent on NMDA receptors (Ekstrom et al., 2001; Burke et al., 2008). Place field expansion occurs in CA3 only during the first experience of a novel path (Roth et al., 2012), but in CA1 it is “reset” after at most 24 h and recurs upon additional exposures to familiar tracks (Mehta et al., 1997; Roth et al., 2012). Place field expansion in CA1 is context dependent, in that upon entry into a novel environment, place fields that form in the novel environment will start as smaller fields even if a different place field from the same cell had undergone place field expansion immediately prior. The new place fields will then undergo expansion in the novel environment (Mehta et al., 1997). Place field expansion is thought to be a result of ongoing synaptic plasticity (Mehta et al., 2000; Yu et al., 2006), encoding the animal’s path(s) into the cellular representation (at least temporarily in CA1). These properties highlight how hippocampal place cell activity dynamically constructs a spatiotemporal framework, within which new experiences are rapidly written into memory while preserving the fidelity and retrieval of old information (O’Keefe and Nadel, 1978; Lisman et al., 2017).

In this study, by analogy, we sought to understand whether PCCs in RSC have properties expected of a memory network, and how these properties compare to hippocampal CA1. Recent studies have illustrated that RSC also forms unique contextual representations (Meenakshi et al., 2021; Subramanian et al., 2024), encodes context-specific task-based features that change over the course of learning (Vedder et al., 2017, Sun et al., 2021), and exhibits separate spatial and rate-based codes for different environments (Miller et. al. 2021; Subramanian et al., 2024). Few studies have compared position correlated activity in RSC and CA1 on the same task (Mao et al., 2017; Alexander and Nitz, 2015; 2017; Subramanian et al., 2024), and none have comprehensively examined the properties of a memory network described above with detailed lap-by-lap analysis of field formation. In this study, we investigated remapping properties of the dysgranular portion of RSC and hippocampal CA1 using two-photon imaging in mice running while head fixed and engaging with visual VR environments.

## 1. Results

### 1.1. 2 P imaging in RSC and CA1 during VR experience

**Fig. 1.**
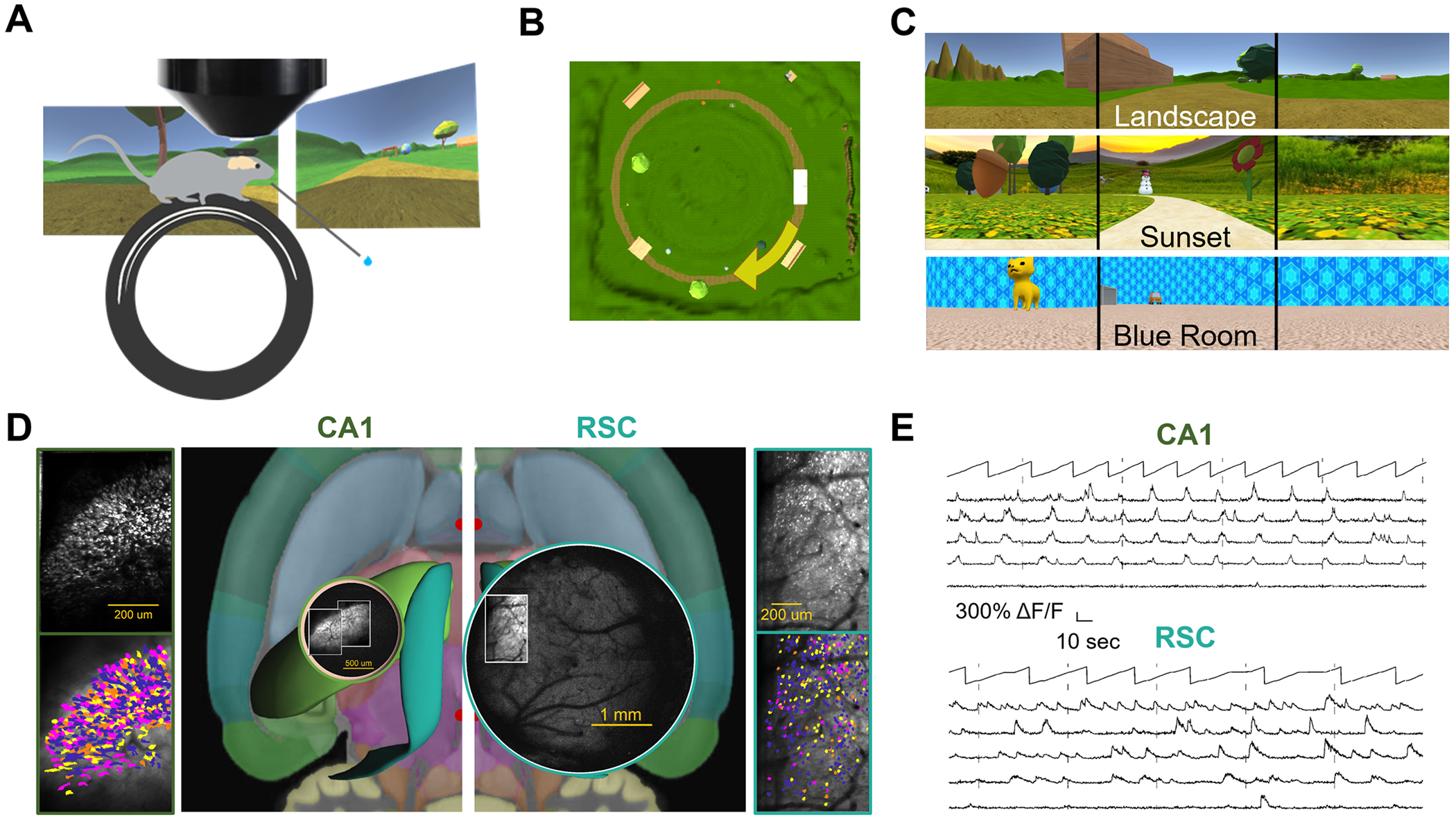
Imaging RSC and CA1 neurons while mice run in virtual reality environments. A. Each mouse was head-fixed over a running wheel, with tablets displaying a VR environment surrounding the mouse from three sides. Cartoon image illustrates the mouse with two tablets (left and front; third tablet, right side, not shown). A reward spout dispensed water or milk rewards. A wide-angled mesoscope recorded neuronal activity above the craniotomy. **B**. Aerial view of the circular VR track environment called “Landscape.” Each lap starts inside the tunnel (white rectangle on the right side of the image) and proceeds clockwise (in the direction of the yellow arrow, which is overlaid on the aerial view). In some different environments the direction was counterclockwise. **C**. Screenshots from three example environments showing views on the left, front, and right tablets. **D**. Transverse mouse brain diagram showing a 3D rendering of retrosplenial cortex (RSC) in teal, and hippocampal CA1 in lime (center; Allen atlas). Overlaid are panoramic two-photon images acquired over either hippocampal CA1 (left circle) or dorsal neocortex (right circle) in separate mice. Regions of interest overlying hippocampal CA1 or RSC were selected within the panoramic view and recorded at 6–8 Hz. Max projection of example imaging sessions over CA1 (top, left) and RSC (top, right) are shown. Bottom right and left show cells detected by Suite2P and manually curated. Neurons are color-coded based on whether they passed position correlation criteria in the familiar (yellow), novel (pink), both (orange), or neither (blue) environments. **E**. ΔF/F activity traces from five example cells in CA1 (top) and RSC (bottom) are shown. Position along the track as a function of time for each session is shown above the activity traces.

To study PCC sequences, we trained mice to run on a simple visual VR task. Mice were head-fixed above a wheel, and viewed visual stimuli on three surrounding tablets (Fig. 1A). Self-induced rotation of the wheel continuously updated the virtual location of the mouse. To motivate running, mice were presented milk or water rewards at hidden reward sites along the track. After 4–6 weeks of training in a single environment (familiar), most mice learned to run consistently over 20 laps in each session.

To study responses in novel environments, several visually distinctive VR environments were created. All were 3–5 m circular tracks with a common 30 cm ‘tunnel’ cue at the start, and various other visual objects on either side of the track. The novel track differed from the familiar in circumference, background images, objects along the track, location of (two) reward sites relative to the tunnel, and sometimes the direction of the curve of the track (clockwise vs. counterclockwise) (Fig. 1B&C). After the animal had been habituated to running in the familiar environment while undergoing imaging (2–5 days), the experimental session commenced. Rest activity (with the wheel blocked from movement) was imaged for 10–30 min before and after each running session, to detect all neurons with any spontaneous activity. Afterwards, the animal was allowed to run for approximately ten minutes in the familiar environment (at least 10 laps). Then, once back at the ‘tunnel’, the mouse was instantly teleported into the novel environment, where it continued running for 15 or more laps.

**Fig. 2.**
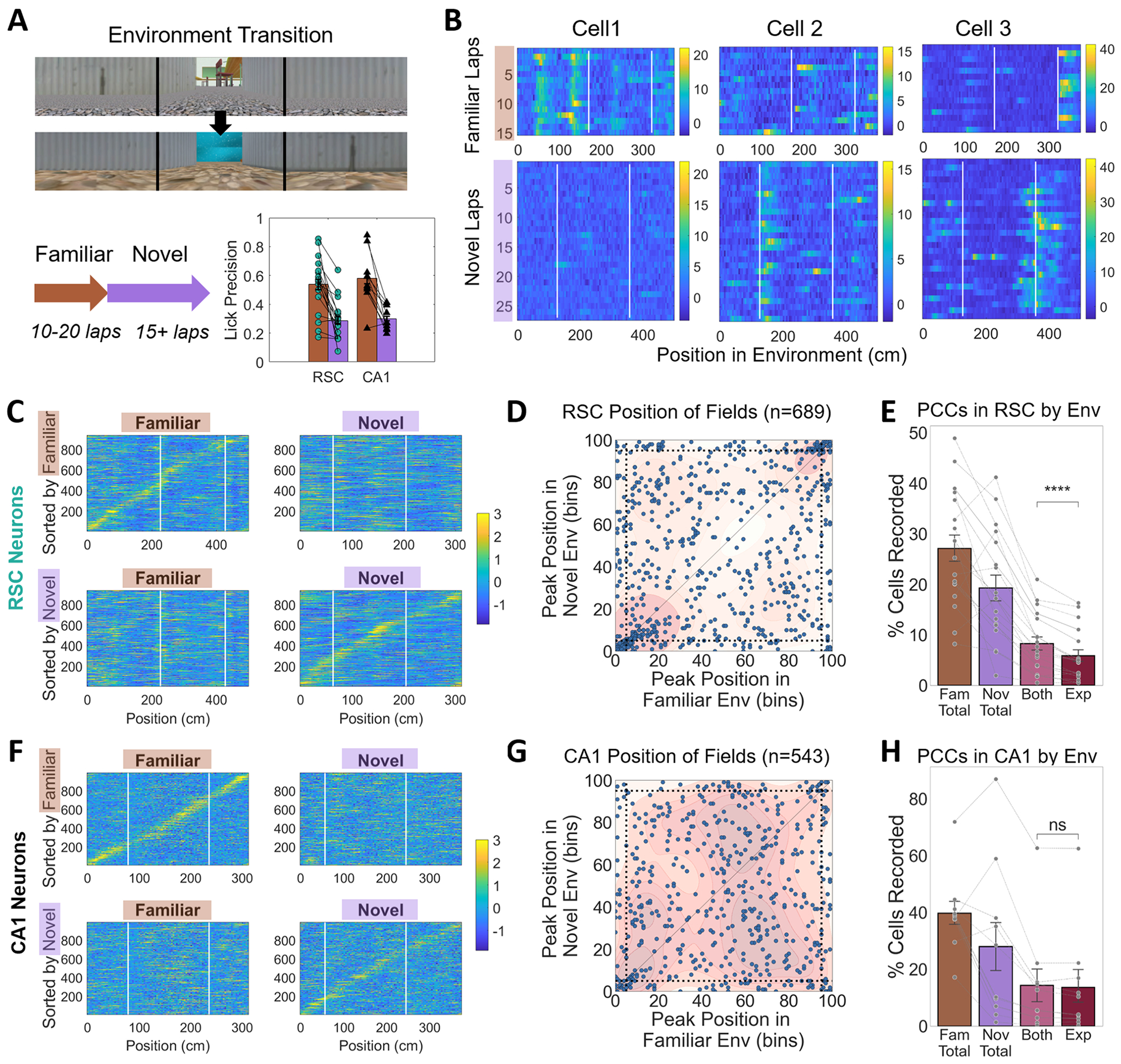
RSC cells exhibit hallmark features of global remapping in novel VR environments. A. Each mouse was trained for 3 + weeks in one of the environments (familiar), and then imaged during a session in which it ran 10–20 laps in the familiar environment and then was ‘teleported’ to a visually distinct novel environment for 20 min (15 + laps). The schematic shows this teleportation protocol. Bottom right shows the licking accuracy of each mouse in familiar and novel environments. B. Position binned activity (ΔF/F) of example RSC cells across laps of the familiar (top) and novel (bottom) environments. Cell 1 established a place field only in the familiar environment, cell 2 exhibited two place fields in the novel environment and none in the familiar, while cell 3 had fields in both the familiar and novel environments but at different relative spatial distances from the tunnel. White vertical lines indicate reward locations along the track. C. Representative data from all RSC cells imaged simultaneously in a single session. Left column shows z-scored, lap-averaged activity in a familiar environment. The right column shows the activity of the same cells averaged across laps in a novel environment. Cells are ordered by the position on the track at which they exhibited peak activity in the familiar environment in the top row, and the novel environment in the bottom row. Activity from half of the final ten laps was removed before averaging and used to determine the order of peak activity. D. For cells in all sessions that had a single field in both environments, the position of peak activity in the familiar versus novel environments is plotted. Dotted lines indicate the extent of the 30 cm virtual tunnel. The background shading indicates the kernel density estimate for the scatterplot. E. The proportion of RSC cells with spatial tuning were tabulated for the familiar (brown), novel (purple), and in both environments (pink). The proportion of cells with fields in both environments was compared to the expected value from a random process with replacement (Exp, dark red). F. G. H. Same as C. D. E. but for cells imaged in CA1. Error bars represent SEM.

To quantify behavioral memory, we measured in which position bins mice licked on each lap. The ten (out of 100) bins just prior to each reward delivery were considered “correct,” and the licks occurring 4 s following reward delivery were excluded (see Methods and Suppl. Fig. S1). Mice licked more accurately in the familiar than in the novel environment (0.553 +/−0.187 vs. 0.291 +/−0.118, paired *t*-test, p < 0.0001). In both environments, mice licked in the pre-reward zone more often than chance (chance=0.2; familiar *t*-test, p < 0.0001; novel *t*-test, p = 0.00034). Once in the novel environment, licks at the former, familiar environment reward locations occurred at no more than chance level (0.211 +/−0.087, p = 0.52). There was no difference in lick performance between mice that were imaged in RSC and mice that were imaged in CA1 (two-way ANOVA, effect of brain region, p = 0.55; Fig. 2A).

Two-photon calcium imaging was used to record cellular-resolution neural activity in RSC or hippocampal CA1 as animals performed the task. In one group of transgenic mice (13 CaMK2a-tTa x tetO-GCaMP6s mice and 3 Thy1-GCaMP6s mice), layer 2/3 excitatory cells were imaged from dysgranular RSC through a glass coverslip (11,485 cells from 18 sessions in 16 mice). In the second group of transgenic mice (6 Thy1-GCaMP6s mice), pyramidal cells in CA1 were recorded through a 1.8 mm cylindrical micro-optic plastic cylinder inserted into a volume of aspirated somatosensory cortex (Supplementary Table 1 shows a list of all analyzed datasets). Recordings yielded hundreds of cells (with at least 1 calcium event) per session (Fig. 1D), from which time-varying calcium signals were extracted (Fig. 1E), aligned to VR and behavioral data, and position binned (see Methods for details).

### 1.2. PCCs in familiar and novel environments

We examined position correlated activity in familiar and novel environments (Fig. 2A). To visualize activity in each session, we separated the data by environment and plotted the lap-by-lap activity of each cell as a function of position (Fig. 2B). To classify cells carrying position information, the same multi-step criterion was applied to the calcium signal from cells in both RSC and CA1 (see Methods for details). In laps in the familiar environment, on average 27.0 % (+/− 11.3) of RSC and 39.7 % (+/− 13.0) of CA1 cells recorded exhibited position-related activity (and thus were classified as PCCs). The average spatial information content (SI) across all cells was calculated to complement this criterion-based approach. The SI was slightly higher in CA1 compared to RSC cells (0.83 +/− 0.08 bits per deconvolved calcium event vs. 0.71 +/− 0.11, t = 3.05, p = 0.005). During the novel environment laps, a substantial number of cells passed the PCC criteria, 19.1 % (+/− 10.7) in RSC and 28.1 % (+/− 26.4) in CA1. The average SI was significantly lower in the novel environment than in the familiar, for both RSC (t = 3.07, p = 0.004) and CA1 (t= 3.03, p = 0.007), and again significantly different between areas (0.73 +/−0.07 in CA1 and 0.61 +/−0.09 in RSC, t = 3.59, p = 0.001). Therefore, although position correlated activity was highest when animals ran in familiar environments, a still sizable number of PCCs (71 % of the familiar environment count) could be identified in both RSC and CA1 on the first day in a novel environment.

### 1.3. Novel environments caused global remapping of RSC activity

To characterize PCC remapping, we examined how the neural activity differed across environments. Cells were ordered based on the position at which their lap-averaged activity peaked in a random subset of the last 10 laps in each environment. Plotting the average of the remaining laps in this order revealed pronounced sequences in RSC and CA1 in both the familiar and novel environments (example in Fig. 2C&F top left and bottom right, respectively).

We looked for hallmarks of global remapping by comparing changes in cell-specific neural activity in familiar and novel environments. To evaluate orthogonality, we applied the order in which cells were sorted in one environment to the lap-averaged activity in the other environment. Rearranging the cells in this way showed that the sequence was not preserved across environments (example in Fig. 2C&F bottom left and top right). These results were consistent across both regions and all animals tested, suggesting that sequences in one environment are unrelated to sequences in another. The hippocampal results confirm that the current VR paradigm can replicate long-standing global remapping results from real-world experiments, while RSC results demonstrate that RSC cells’ activity changes in a manner consistent with global remapping.

Global remapping requires that even cells active in multiple environments would have uncorrelated activity patterns. For cells that had a single field in both environments, the locations of those fields across environments were essentially uncorrelated in CA1 (r = 0.08, p = 0.06; Fig. 2G; Table 1). In RSC, there was a small but significant correlation between field locations (r = 0.28, p < 0.001; Fig. 2D; Table 1) driven primarily by cells with fields near the common ‘tunnel.’ Excluding just those fields (within five bins of the beginning or end of the track, marked by dotted lines) reduced the correlation between remaining field locations in RSC cells (r = 0.14, p = 0.003; Table 1). Thus, for most of the track, there was little relationship between where the cell would establish fields in the novel and familiar environments.

Efficient pattern separation also requires that each environment be encoded by an uncorrelated subset of cells. If position-correlated fields are allocated randomly and all imaged cells are equally likely to form a field (this is known to be false: pyramidal cells have different excitabilities, and thus different likelihoods of activation/allocation (Rich et al., 2014; Witharana et al., 2016); but in small environments, this assumption can be useful; see also Suppl. Fig. S2), the expected proportion of cells with position-correlated fields in both environments should be the proportions of PCCs in each of the environments, multiplied together. Indeed, we found that the real proportion of cells with fields in both environments was close to the mathematically expected value. The real proportion of RSC was significantly but weakly higher than expected (2.41 % higher; Fig. 2E; Table 1). Removing cells with fields near the tunnel reduced the RSC difference from expected (1.77 % higher) but it remained significant. Thus, in RSC, cells with fields in one environment were only slightly favored to have a field in another. The real proportion of CA1 cells was not significantly different from expected (0.72 % higher; Fig. 2H; Table 1).

### 1.4. Population activity changed immediately upon entry into the new environment and then evolved into a new stable representation

**Fig. 3.**
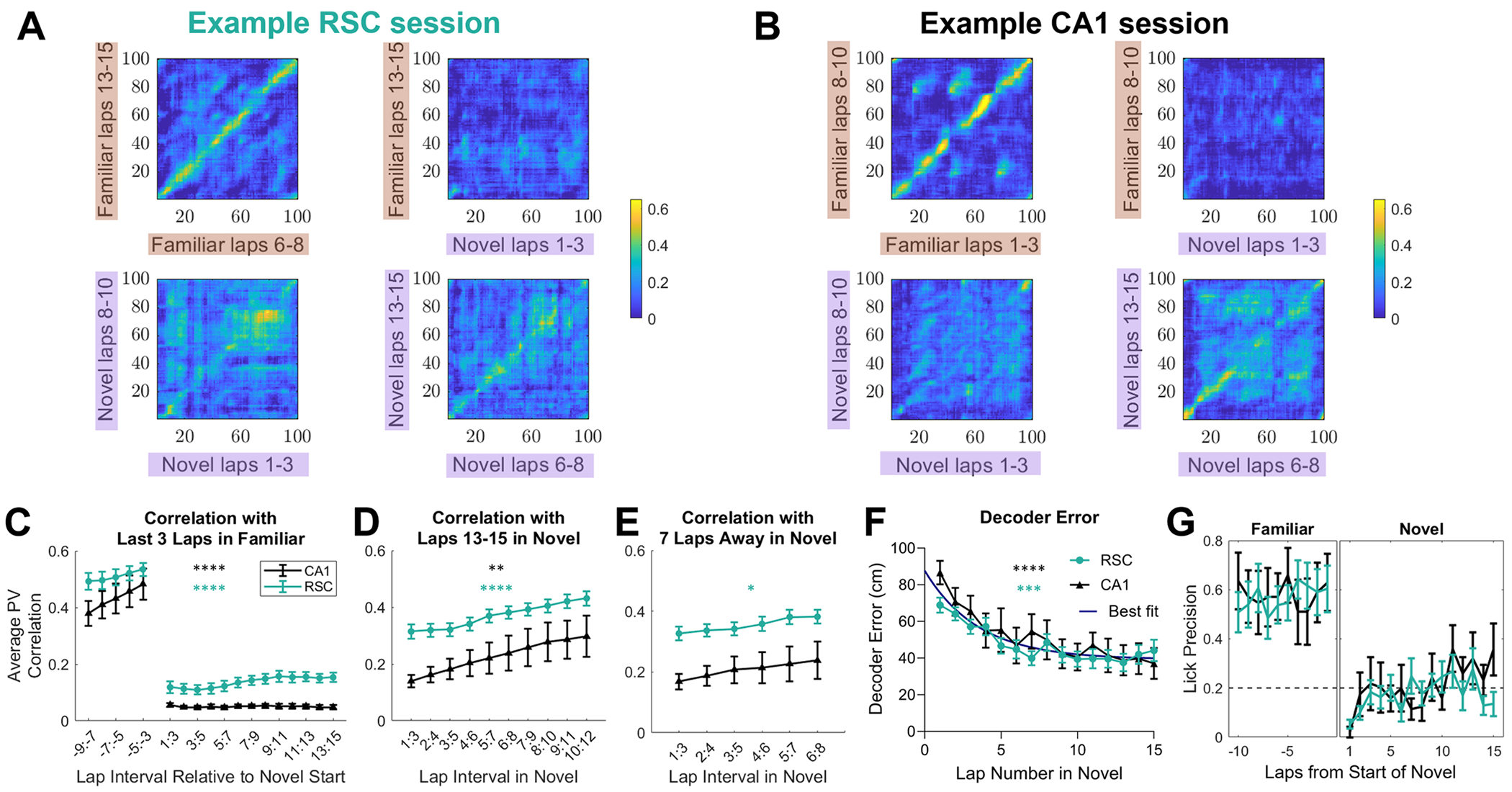
Population vectors show an abrupt change at the switch in environments, and then a gradual stabilization of population activity in the novel environment. A. The activity of all simultaneously imaged RSC neurons during an example session in each position bin (the PV), averaged across a 3-lap interval, was correlated with all position bins during a different 3-lap interval. Top row shows the correlation of late familiar environment laps with early familiar environment laps (left) and early novel environment laps (right). Bottom row shows the correlation of late novel environment laps with early novel environment laps (left), and middle novel environment laps (right). Familiar laps were chosen such that the last lap used is the last lap in the familiar environment (which differed across mice), and the first lap used is 10 laps before that. B. Same as A, except using CA1 cells imaged in a different mouse. C-E. The PV for each spatial bin in each 3-lap interval was correlated against the same spatial bins in a reference set of laps (reference laps are noted in the figure title). The average across bins was taken as the correlation for each set of laps (i.e. averaging along the diagonal in the correlation matrices shown in A). C. The reference laps were the final three laps in the familiar environment (here numbered −2:0), and were correlated with every non-overlapping lap interval before and after the transition to the novel environment. The example at the top left of A and B is in the −9:-7 data point, and the top right is 1:3. D. The correlation between late laps (13–15) in the novel environment to all non-overlapping earlier lap intervals. E. Each lap interval was correlated with an interval 7 laps later. The first data point is laps 1–3 correlated with laps 8–10 as shown in the bottom left of A and B, and the last data point is laps 6–8 correlated with laps 13–15, as shown in the bottom right of A and B). F. The difference between actual position and decoded position in each novel environment lap. Bayesian decoder trained on all laps but one was used to decode the position of the left-out lap. The number of neurons used to decode position was matched across datasets. Error bars represent SEM. Asterisks indicate that the dependent variable was significantly correlated with laps for RSC (teal), or CA1 (black) datasets. G. Most mice stopped licking in the first lap of the novel environment, and then gradually started learning the new reward locations. Dotted line indicates chance level licking. When mice did not show any anticipatory licks in a lap at all, their performance was counted as zero.

The substantial fraction of PCCs in the novel environment suggests that both regions could form a representation of the environment within several minutes. To understand the dynamics of PCC sequence formation, we first examined how the activity changed in the population. We calculated a population vector (PV) at each position bin of the environment, averaged over a sliding window of three consecutive laps. These PVs gave the state of the neural network at a specific bin for a specific lap interval. The PVs at the final (or late) laps in each environment were then correlated against PVs in other lap intervals. Early laps in the familiar environment were highly correlated to final laps, suggesting that the network rapidly entered a stable state when presented with well-trained inputs (example in Fig. 3A&B top left). As expected from the global remapping findings, familiar laps showed no correlation to novel laps (Fig. 3A&B top right).

PV correlations between each bin and the corresponding bin in a different set of laps were averaged (i.e. along the diagonal in Fig. 3A&B). Correlating the last 3 laps in the familiar environment with all other non-overlapping sets of 3 laps showed that remapping occurred immediately upon entry into the novel environment (Fig. 3C; Table 1). Correlating late laps (13–15) in the novel environment with previous novel environment laps showed a gradual rise in similarity (Fig. 3D; Table 1; see also Suppl. Fig S3). To check if this steady rise was due to gradual drift across laps (in which case laps distant in time would be less similar to adjacent laps, regardless of novelty), or to an increasing stabilization of population representations, each lap interval was compared to an interval 7 laps later (Fig. 3E; Table 1). RSC showed a significant increase in stability across laps. CA1 datasets showed a lot of variability, and thus we were not able to show a significant effect of laps, even though some datasets clearly showed stabilization of representations (Fig. 3B and Suppl. Fig. 3 A). We compared CA1 and RSC, and found no difference other than a difference in intercept (see Table 1). In sum, these data show that, in a novel environment, RSC population activity immediately de-correlates from that in the familiar environment, and then a new representation stabilizes across subsequent laps.

To further determine the stability of the neural representations of novel environments, we used a Bayesian decoder to predict the position of the mouse from RSC or CA1 activity in each lap, using a decoder trained on every other lap (leave one out cross validation; see Suppl. Fig. S4). In each dataset, we ran the decoder on a random subsample of 146 neurons (the number in the smallest dataset) 100 times and averaged the results to get a final decoder error. There was no difference in the mean decoder error between RSC and CA1 in either familiar (RSC: 18.9 +/− 13.1 cm; CA1: 14.5 +/− 8.2 cm) or novel (RSC: 44.9 +/− 15.0 cm; CA1: 45.1 +/− 20.0 cm) environments (see Suppl. Fig. S4D, Table 1). The decoder error was higher in the novel environment compared to the familiar environment, even when considering only the best 5 laps of the session (RSC: familiar 10.3 +/− 9.7 cm; novel 25.1 +/− 14.4 cm; CA1: familiar 5.6 +/− 2.3; novel 24.4 +/− 18.8, Table 1). Decoder error decreased significantly across laps in both CA1 and RSC datasets (Fig. 3F; Table 1). This decrease was exponential, showing a plateau after around 10 laps (Table 1). However, the decoder error continued to decrease further on subsequent days (Suppl. Fig S4E). There was again no difference in decoder error evolution across laps between brain regions.

Behaviorally, most mice (20/28 sessions) stopped licking in anticipation of rewards during the first lap in the novel environment. Many mice had resumed anticipatory licking by lap 3 (20/28 mice), but their licks were not restricted to the pre-reward zones. Licking accuracy increased slightly on subsequent laps, but remained much lower than in the familiar environment (Fig. 3G, Table 1).

### 1.5. In-field activity increased during novel field formation

**Fig. 4.**
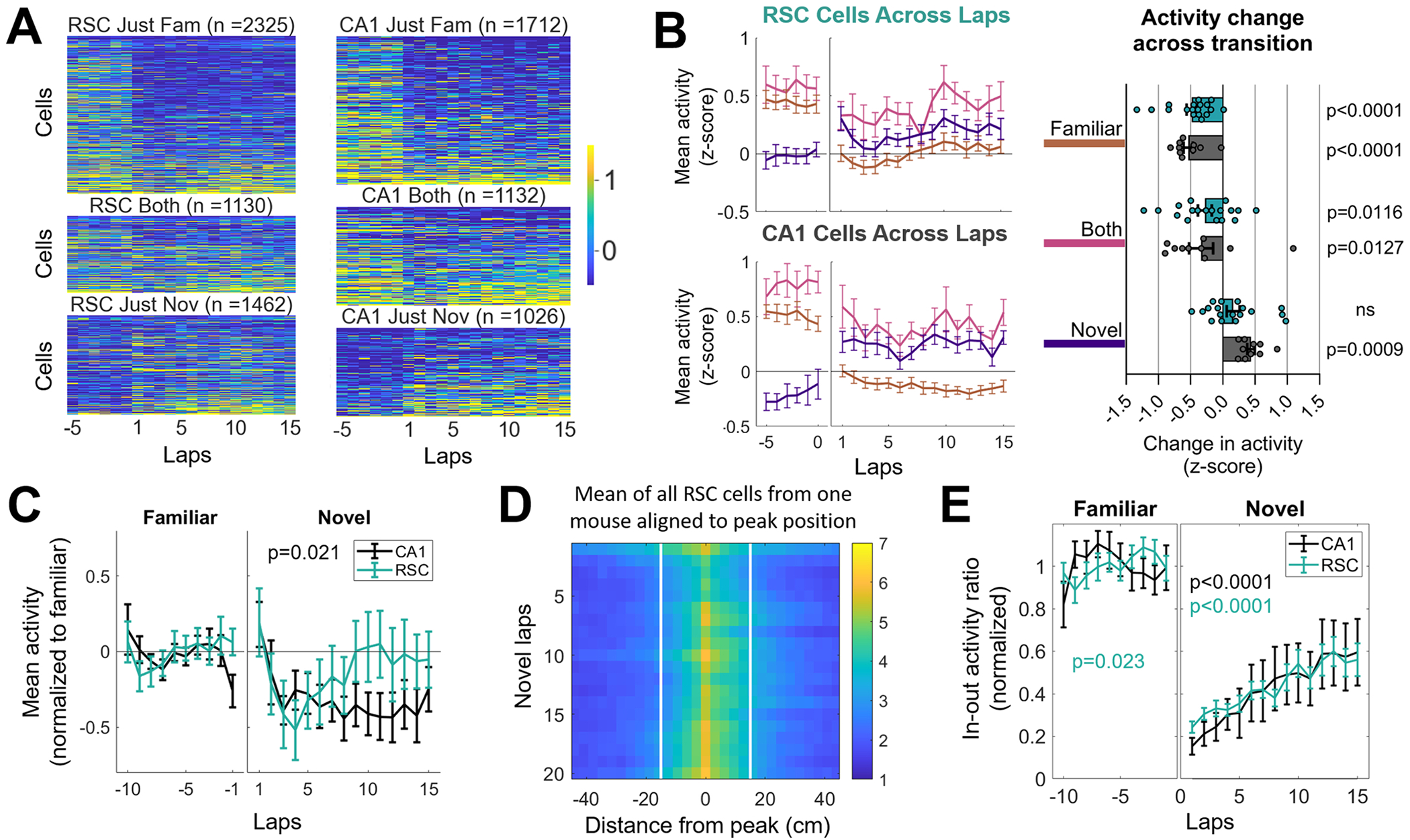
Mean activity and activity within position fields tunes up over the course of a few laps in a novel environment. (A). Lap averaged Z-scored calcium activity for all recorded PCCs with place fields in just the familiar (top), both (middle), or just novel (bottom) environments (RSC left, CA1 right) for laps surrounding the environment transition. Activity across the whole lap is averaged. Cells are ordered by mean activity in the final laps in the novel environment (for most sessions this was later than lap 15). B. Average z-scored activity of all cells with place fields in the familiar, novel, or both environments. Activity is averaged across all cells in a session, and then across mice, and plotted by individual laps (left), or the average of the last 3 laps in familiar versus the first 3 laps in novel (right). C. Z-scored activity averaged across all cells (not just PCCs as in B), showed that activity in CA1 went down across laps in the novel environment. D. Example from a single RSC session showing all cells aligned to their peak position. Aligned activity was averaged for each lap. White lines indicate the 30 cm region which was considered “in field.” E. A ratio was computed to quantify how activity within the field evolved relative to activity outside the field. The ratio was then normalized to the mean ratio in the familiar environment for each mouse.

Next, we investigated how overall activity levels fluctuate around laps surrounding the environment transition. To evaluate changes in average PCC firing rates in meaningful neural subpopulations, we grouped cells across sessions according to whether they exhibited fields in just the familiar, just the novel, or both environments. Neural activity from cells in each category were averaged by lap then z-scored by session. Unsurprisingly, neurons showed high activity in whichever environment the cell had fields (Fig. 4A&B), and low activity in environments in which they lacked a position-correlated field (Fig. 4B). Consequently, activity of PCCs in RSC and CA1 with fields in just the familiar environment significantly decreased once the animals entered the novel environment, and cells with fields in just the novel environment increased across the environment transition. The change in activity of PCCs with fields in just the novel environment was a bit smaller (and not significant for RSC). Interestingly, cells with place fields in both environments (in both RSC and CA1) showed a significant drop in activity after the environment transition. This matches with the findings in Knierim et al. (1998), in which CA1 place cells with fields in both configurations decreased their activity in the several minutes after global remapping in response to a cue manipulation, and then gradually rebounded. The mean activity across the whole population of RSC cells did not change across the environment transition, but CA1 activity did decrease significantly with laps in the novel environment (Fig. 4C).

Next, we analyzed the development of spatial activity, by aligning the activity of all cells to their peak activity bin. Plotting the average PCC field activity for each lap in the novel environment showed that infield activity was low in the first several laps (Fig. 4D). We quantified this by calculating the ratio between in-field (within a 30 cm region surrounding the peak) and out-of-field (all other position bins) activity for all cells and then normalizing to the mean in-out ratio in the familiar environment (Fig. 4E). The in-to-out field activity ratio increased over the course of the first several laps, for both RSC and CA1 (Table 1). Although the activity across cells correlated with running speed (Suppl. Fig. S5B), and the running speed did change across laps in the novel environment (Suppl. Fig. S5C; Table 1), these effects were small, and could not account fully for the changes in mean activity, in-out ratio, or the between-lap correlations across laps 1–15 (Suppl. Fig. S5E–I). There was no change in stopping time or acceleration across laps in the novel environment (Suppl. Fig. S5J–L). Taken together, these findings show that the gradual increase in population decoding of position information is accompanied by an increase in activity of PCCs with fields in the novel environment, and an increase in in-field activity. There was no difference between RSC and CA1 in the rate of increase of these measures, suggesting that spatial information is expressed in both regions right from the beginning of a new experience.

### 1.6. Experience-dependent backwards place field shift did not occur in RSC

**Fig. 5.**
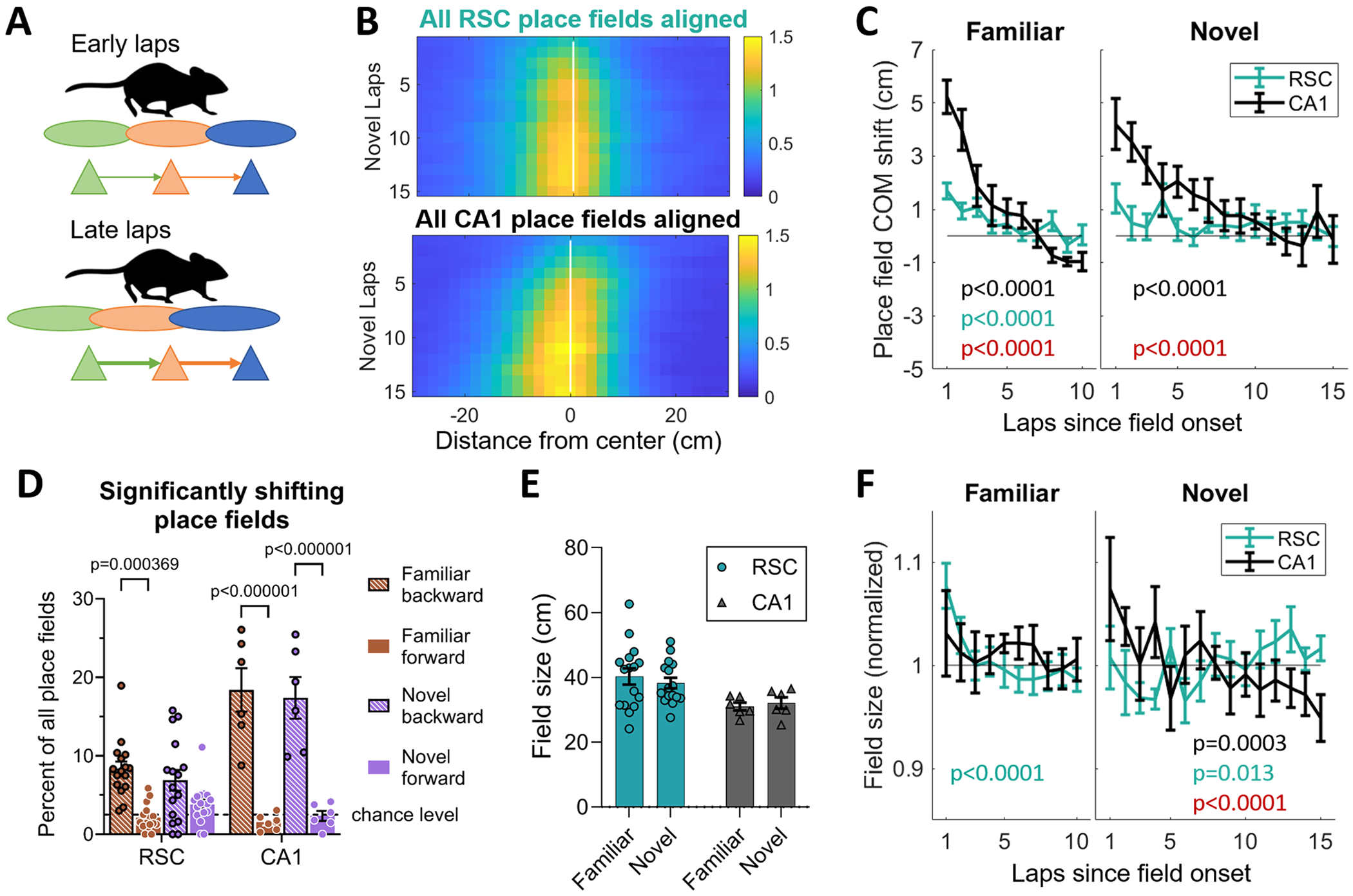
Place field expansion and center of mass (COM) shift. A. Schematic showing hypothetical place field expansion and center of mass shift from early to late laps, and the theorized mechanism. In early traversals of an environment, CA1 place cells (triangles) which express place fields at various locations along the track (ovals) are weakly connected. Across traversals along the same path, place cells with adjacent place fields strengthen their connections asymmetrically, causing post-synaptic cells to fire earlier, and expanding the place fields in the backwards direction. B. All RSC (top) and CA1 (bottom) place fields that were identified across mice were aligned to their peak location, and their deconvolved calcium events were averaged for each lap, for illustration of place field changes across laps. C. The COM was calculated for each place field by weighting each position bin within the boundaries of the field with the number and amplitude of calcium events in that bin. Then the COM was calculated for each lap with at least two deconvolved events, and subtracted from the mean COM for that field. The COM of all fields identified in a single session were averaged, before averaging across mice. CA1 cells showed a COM shift similar to previously published data. RSC fields showed a significantly smaller COM shift than CA1 in the familiar environment, and no significant shift in the novel environment. D. For each place field, we evaluated if there was a significant difference between the COM on laps 1–3 and all remaining laps (t-test, α<0.05). Significant place fields were classified as backward or forward shifting. Marked p-values indicate a significant difference between the number of forward shifting and backward shifting fields in each dataset (Sidak’s multiple comparisons test). E. Field size was calculated for each place field by measuring the distance between the first and last deconvolved calcium event on each lap, and then averaging across laps. There was no significant difference between RSC and CA1 place field sizes. F. For each lap, the field size was normalized to the average across laps. Laps with fewer than two DCEs were excluded from analysis, same as in C. Error bars represent SEM. P-values indicate a significant correlation between the dependent variable and lap number for RSC (teal), or CA1 (black) datasets, or a significant difference between the slopes of RSC vs. CA1 datasets (red).

A major effect of repeated route following in the hippocampus is the phenomenon of backwards shift and expansion of place fields, which is thought to indicate asymmetric NMDA-dependent plasticity between neurons representing sequential positions (Fig. 5A; Ekstrom et al., 2001). CA1 place cells show backwards (opposite to direction of motion) shift and expansion, in both familiar and novel environments (Mehta et al., 1997; Dong et al., 2021). To check if this phenomenon might also occur in RSC or be transmitted to RSC from hippocampus, we calculated the center of mass (COM) of each field on each lap, using deconvolved calcium events (DCEs, see Methods). Because fields were not expressed on every lap, especially in the novel environment, only laps with significant in-field activity (at least 2 DCEs), starting with the onset lap for each field, were considered. As in previous studies, CA1 PCC fields showed a backwards COM shift in the familiar environment (Fig. 5C left; Table 1). RSC PCCs showed a significantly smaller backwards shift (Fig. 5C left; Table 1). CA1 fields in the novel environment also showed a significant backwards COM shift, and RSC fields did not (Fig. 5C right; Table 1).

To determine if individual fields in RSC showed COM shift, we compared the COM in laps 1–3 with all other laps for each field, and determined if there was a significant difference (*t*-test, p < 0.025). Approximately 20 % of CA1 fields and 8 % of RSC fields showed a significant backwards shift (Fig. 5D). In both brain areas, the number of fields showing forward shift was at chance level. While the number of RSC place fields in the familiar environment showing backwards shift was significantly higher than the number showing forward shift, it was significantly lower than the percentage in CA1 (Table 1).

To measure place field expansion, place field size was calculated. Lap-averaged place field sizes were not different between familiar and novel environments, and RSC place fields were 19 % bigger than CA1 place fields (Fig. 5E). For each lap (with at least 2 DCEs), the distance between the first and last calcium event was calculated. There were only small changes in place field size across laps, in both brain regions (Fig. 5F; Table 1). However, when we measured place field size by using fluorescence activity (ΔF/F) instead of deconvolved calcium spikes (by measuring the width at half amplitude of the peak in each lap; see methods), we found a significant place field expansion in CA1, but not in RSC (see Suppl. Fig S6).

Thus, we confirmed that we were able to measure COM shift across laps in CA1, in both familiar and novel environments using calcium imaging, both by looking at deconvolved events, and fluorescence activity (see Suppl. Fig S6). RSC place fields showed a much smaller (if any) average backwards shift, and in fewer neurons. Lap-wise place field size proved to be more difficult to measure using calcium activity, with different methods showing opposite results. In conclusion, although RSC place fields appeared and developed with similar time dynamics to CA1 fields, they did not show the hallmarks of NMDA receptor-dependent sequential coupling to the same extent as is observed in hippocampus.

### 1.7. Accuracy of licking behavior was correlated with neural coding of space

**Fig. 6.**
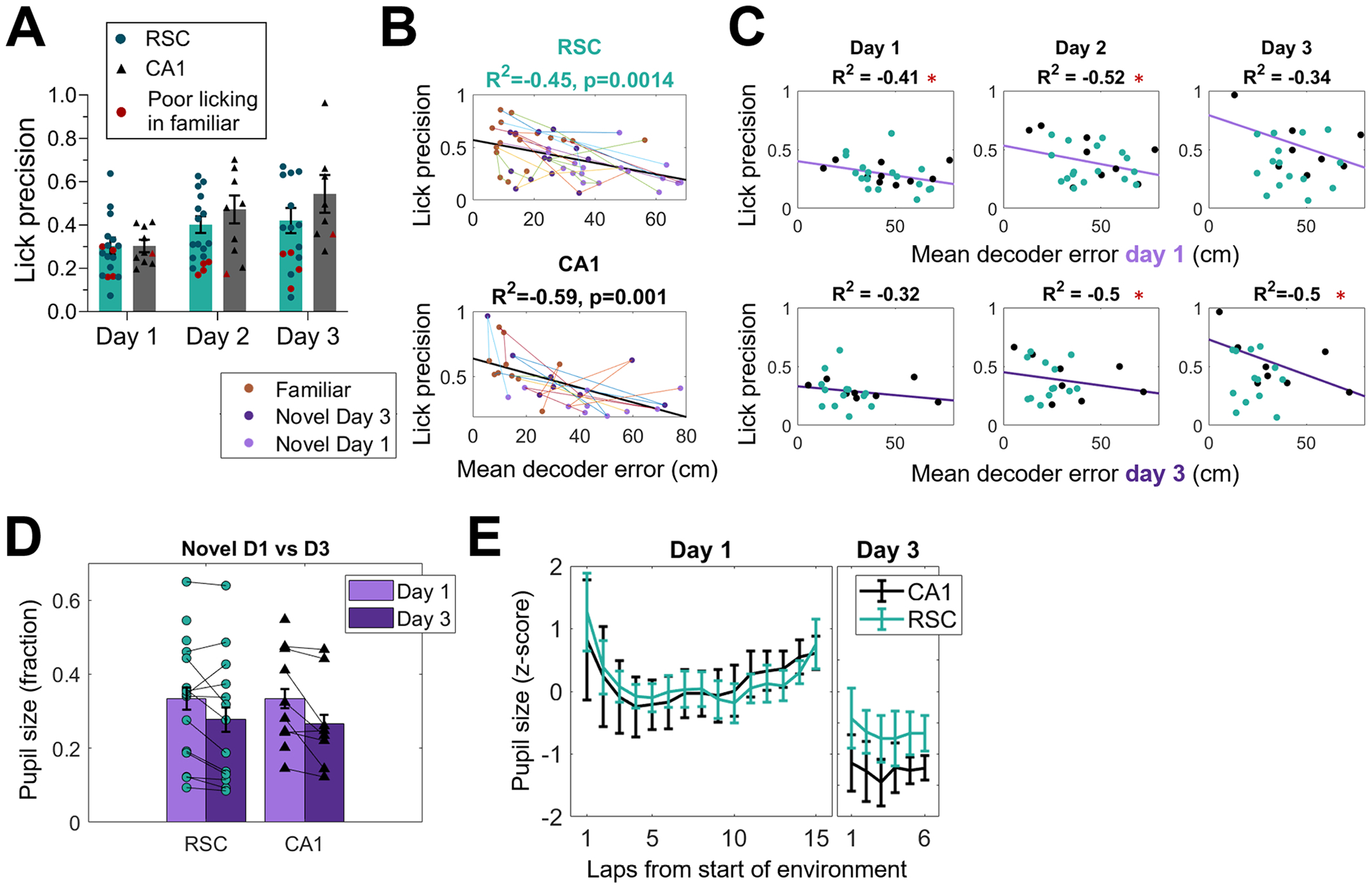
Changes in behavior as mice learn new environments. A. Lick precision improved across days. A mixed effects analysis showed a significant effect of days (p = 0.0002), and no effect of which brain region was imaged (p = 0.12). Mice that had poor licking performance in the familiar environment (<0.4) in the session prior to novel environment exposure are marked in red. These sessions were excluded from the analysis in C on the inference that their poor performance was not due to lack of environment knowledge, and instead showed a lack of understanding of the task. B. To determine if behavioral performance was related to neural activity, we correlated the lick precision with the decoder error in the same session. To get a full range of behaviors, we pooled familiar, novel day 1, and novel day 3 sessions. Sessions coming from the same mouse are connected by lines with matching colors. The black line indicates the best linear fit (least squares). Both RSC (top) and CA1 (bottom) showed a significant negative correlation between decoder error and reward anticipation. C. We correlated lick precision on days 1–3 in the novel environment (same data as in A, but without the red points) with decoder error measured on days 1 (top row) and 3 (bottom row). RSC (teal dots) and CA1 (black dots) datasets were pooled in this analysis. Significant correlations are marked with red asterisks (* = p < 0.05). D. Pupil size in the novel environment (as a fraction of the size during rest in darkness) was larger on day 1 than on day 3, suggesting that the mice responded to novelty by increasing their arousal. E. Pupil size was often high in the first lap in the novel environment, rapidly decreased, and then increased gradually across subsequent laps. This pattern was not displayed by all mice, however. A two-way ANOVA showed a significant effect of laps on pupil size (p = 0.0042) on day 1, and no effect of imaged brain region (p = 0.9). There was no significant effect of laps on day 3, on which the average diameter was overall reduced.

Most mice were exposed to each novel environment for three days. The mice’s accuracy in anticipatory licking improved across days (Fig. 6A), but varied greatly across mice. Thus, we were able to correlate the accuracy of neural activity (decoder error) with the accuracy of licking behavior across mice to study how neural activity and behavior were related. To get a full range of behaviors, we initially pooled familiar, novel day 1, and novel day 3 sessions (Fig. 6B). The average decoder error measured in both RSC and CA1 showed a highly negative correlation with lick precision in the same session (RSC: R^2^ = −0.45, p = 0.0014; CA1: R^2^ = −0.59, p = 0.0010). However, some sessions with low decoder errors still showed low lick accuracy. On the other hand, few sessions with high decoder error had high lick precision, indicating that neural coding of position may be necessary for high lick precision, but is not sufficient.

Further, we analyzed whether spatial coding accuracy could predict licking behavior on prior or subsequent days. Because there was no difference between decoder errors measured in CA1 and RSC, and both were correlated with within-session lick accuracy, we pooled data from both regions for this analysis. Additionally, we removed datasets from mice which had poor accuracy in the familiar environment, under the assumption that these mice may have had trouble understanding the task, rather than the spatial context. Decoder performance on day 1 was correlated with licking accuracy on both days 1 and 2, but interestingly the correlation with day 2 was higher (Fig. 6C, top row; day 1: R^2^ = −0.41, p = 0.048; day 2: R^2^ = −0.59, p = 0.012). This may indicate that neural coding of position in CA1 and RSC precedes behavioral accuracy. Decoder error on day 3 was not correlated with behavior on day 1, but was similarly correlated with behavior on days 2 and 3 (Fig. 6C bottom row: day 1: R^2^ = −0.32, p=0.21; day 2: R^2^ = −0.50, p = 0.039; day 2: R^2^ = −0.50, p = 0.038). All of these correlations are rather weak, despite a large number of mice, and thus factors other than neural activity in RSC and CA1 must play a role in this behavior.

Finally, we analyzed the changes in pupil size across time in the novel environment (Fig. 6D&E). In addition to luminance changes, arousal levels and other cognitive processes influence pupil size. By averaging pupil size across a full lap in each environment, and only comparing laps within the same environment, we controlled for luminance effects on pupil size. Pupil size was larger on day 1 than on day 3 (Fig. 6D; Table 1), confirming that novelty increased arousal and pupil size. Pupil size changed across laps on day 1 (a repeated measures ANOVA showed an effect of laps; Table 1; Fig. 6E), however this effect was not linear (a simple linear regression was not significant). We noticed two main trends in the pupil size changes: an increase in pupil size during the first lap in a novel environment, which went away within a couple of laps, and a more gradual and steady increase in pupil size across laps, continuing for the entire session. The first effect was observed only on day 1, and the second effect was observed on subsequent days as well. These trends were explored further in Supplementary Figure S7. We analyzed changes in other pupil parameters, such as speed, variance in horizontal movement and number of saccades, but none of these showed large consistent changes across laps or between environments (Suppl. Fig. S7F–I). In conclusion, novelty was associated with several behavioral changes, some of which may be useful in understanding the neural processes underlying memory formation.

## 2. Discussion

We found evidence that position correlated activity develops in hippocampus and RSC on a similar timescale. While the activity in RSC was somewhat less spatially tuned (the spatial information, ratio of infield to out of field activity, and percentage of cells showing spatial tuning were all lower), across the population the amount of spatial information was the same (we were equally able to decode spatial information by using RSC as by using CA1 activity), and the time course of the expression of spatial information in a novel environment was remarkably similar. The main differences we found between RSC and CA1 were subtle: a difference in the amount of remapping near one prominent object (the tunnel) shared across environments, and a reduction of experience-dependent place field expansion in RSC. We also determined that RSC activity, like hippocampal activity was modulated by running speed, however this modulation was not large enough to explain the activity changes related to novelty that we observed.

A major question raised by these results is why activity is so similar between RSC and hippocampus. According to memory index theory (Teyler and DiScenna, 1986; Teyler and Rudy, 2007) information about attributes (memory content), in neocortex, gets linked to memory indices in the hippocampus. However, there are not enough synapses between hippocampus and neocortex to permit direct transmission of sparse CA1 representations to association cortex neurons. Therefore, the index signal must first get compressed (in subiculum), and then extracted back into a sparse code in the neocortex (Barnes et al., 1990; McNaughton, 2010; Kim et al., 2012; Witharana et al., 2016; Kitanishi et al., 2021). We posit that PCC activity in RSC is a representation of that memory index and distributes spatial information further into neocortex (Kaefer et al., 2022).

In contrast to our results, in freely moving animals, RSC does not exhibit place fields as similar to those in CA1 as is shown here (Alexander and Nitz, 2015; 2017; Subramanian et al., 2024). Compared to dorsal CA1 place fields, RSC fields were found to be much larger (at least an order of magnitude larger), less sparse, show lower spatial information content, and be modulated by factors other than position (such as head direction and acceleration). In freely moving rodents on narrow tracks, some RSC cells did show smaller place fields compared to two dimensional environments (Subramanian et al., 2024; Alexander and Nitz, 2017), but not as many as in our experiment. Place field size in hippocampus is influenced by many factors including recording location along the dorso-ventral axis of CA1 (Jung et al., 1994; Maurer et al., 2005), environment size (Muller and Kubie, 1987), cue density (Battaglia et al., 2004), self-movement as determined by the vestibular system, proprioception, and optic flow (Terrazas et al., 2005), and the gain of the self-motion signal (i.e. how much the self-motion signal influences each brain region; Jayakumar et al., 2019). Some of the difference in field size between our study and other RSC recordings can be accounted for by the differences in the experiments. For example, almost all of our recordings were in superficial agranular RSC, whereas other experiments recorded spikes in deep layers of granular RSC (Subramanian et al., 2024; Alexander and Nitz, 2015; 2017). Additionally, head-fixed animals do not experience head rotation and acceleration, which normally contribute to self-motion information; however, this should result in larger place fields compared to real-world environments (Terrazas et al., 2005). The lack of head rotation also prevents us from analyzing head-direction selectivity by RSC cells, but again, if head-direction were a large part of RSC selectivity in our study, we should see fewer cells with small fields, not more. On the other hand, if mice can use the optic flow in our VR to trigger the perception of body rotation, then RSC cells could be responding to this allocentric (relative to the VR environment) body position, instead of (or in addition to) distance, and we have no way of disambiguating the two in this experiment. However, experiments with virtual linear tracks (and teleportation between start and end) find similar results to ours (e.g. Mao et al., 2020, Fischer et al., 2020). It is likely that other differences between VR and real-world environments contribute to the difference in RSC field sizes. Notably, visual virtual environments tend have cues that are closer to the track than real world tracks, prompting the animals to rely on proximal cues, rather than distal room cues for navigation. These pass through the visual field faster and thus may prompt faster changes in cell activity. Additionally, VR environments tend to have a higher density of visual cues than real world tracks, and cue density is known to impact place field size in the hippocampus (Battaglia et al., 2004) and cortex (Burke et al., 2012).

The most compelling reason for the differences between freely moving and head-fixed treadmill running studies is that RSC can respond flexibly to a wide variety of cues (for review see Smith et al., 2018). In our task, there are many constant variables (e.g. head position and angle, smells, tactile cues), and yet the RSC was able to respond variably, presumably using the few varying cues that were available. This did not appear to be reward-driven, as RSC activity could decode spatial distance well before mice showed accurate reward responses. It also did not require a lot of learning, since spatial tuning developed within several trials. However, the sensitivity of RSC neurons to visual VR cues (and spatial distance) despite other cues being constant likely did require learning, as our mice were extensively trained in the familiar environment, while other VR studies with less training showed a slower development of place fields (Fischer et al., 2020). The relatively small place fields and high spatial information in RSC could be due to a higher reliance on relatively denser visual cues in VR experienced mice, or a larger influence of spatial signals from hippocampus, given a lack of variance of other signals (e.g. head-direction). Our experimental design did not allow us to determine which of these was the case.

Hippocampal lesion experiments have shown that, post-lesion, RSC place fields do not form as well in novel environments, but familiar environments retain PCCs after the lesion (Esteves et al., 2023). This is consistent with behavioral experiments that show that over time, memories that initially depend on hippocampus are consolidated into hippocampus-independent form in neocortex (Tse et al., 2007; 2011; Cowansage et al., 2014). Our data are consistent with a model in which the hippocampus and retrosplenial cortex interact closely during the formation of novel environment representations.

The reduction in backward expansion of RSC PCC place fields, however, raises a major question. Is hippocampal activity merely permissive of spatial tuning development or is CA1 activity somehow transferred directly to RSC? If the latter, then the lack of transfer of the expanded component of the CA1 place fields must be accounted for. Communication between hippocampus and RSC during active behavior occurs via a temporal structure defined by the theta rhythm (4–12 Hz). The local field potential in both areas oscillates nearly synchronously at this rhythm during locomotion (Alexander et al., 2018). Many RSC neurons are phase locked to this rhythm, and the majority of those fire at the theta trough or ascending phase (180–300 °; Alexander et al., 2018; 2020). The firing rates of CA1 and subiculum neurons peak at the theta trough (~180 °), just before RSC. However, hippocampal neurons are not phase locked, instead, they “phase precess” as the animal passes through their place fields. As an animal enters a cell’s place field, that cell fires in late (descending) theta phases, in the middle of the field the cell fires bursts of spikes around the trough of the theta cycle, and just before the animal exits the field, spikes occur early in the theta cycle (ascending phase; O’Keefe, Recce, 1993; Skaggs et al., 1996). When a place field shows experience-dependent backwards shift/expansion, the added spikes occur almost exclusively in late theta phases (during entry into the field). Thus, RSC neurons which are tuned to the hippocampus in the early or middle phases of theta would not show as much shift/expansion. While there is evidence that groups of neurons can “tune in” to different brain regions at different phases of theta (Colgin et al., 2009), it is as of yet unknown at which phases RSC and CA1/subiculum might communicate. Theta phase locked interneurons in RSC might implement this “gating.” Synaptic communication is fast relative to the theta rhythm (~2 ms per synapse, thus 4–6 ms from CA1 to agranular RSC, or ~3–5 % of a theta cycle), but it might take significantly longer to produce a responding action potential if integration of several EPSPs from distal dendrites is required. Thus, it is possible, but not necessarily true, that ascending phase RSC spikes are a result of ascending phase CA1 spikes. Instead, the offset in peak firing rates might indicate that RSC neurons firing on the ascending phase might be responding largely to activity in CA1 and subiculum at theta trough (Alexander et al.,2023). Additionally, different RSC neuron types show phase locking to different phases of theta. In particular, egocentric boundary cells were found to fire principally on the descending phase (Alexander et al., 2020). Further studies are needed to determine if the small subset of RSC cells we found which show place field shift show theta phase locking at a different phase than the non-shifting cells. Another possibility is that another brain region, such as entorhinal cortex, contributes spatial information to both RSC and CA1, and hippocampal activity only indirectly influences spatial tuning in RSC.

Much is still to be learned about how exactly novel representations form and the direction(s) of information flow, but we have shown that this endeavor will require the consideration of multiple brain regions, and careful analysis of an animal’s past learning. Nevertheless, the rapid establishment of position correlated neural activity in neocortex suggests that memory formation and its consolidation may involve the coordinated transfer of memory indices from hippocampus to neocortex.

## 3. Methods

### 3.1. Experimental model and subject details

#### 3.1.1. Animals

All animal protocols and procedures were conducted with the approval of the Animal Care and Use Committee at the University of California, Irvine. Transgenic mouse lines expressing GCaMP6s in excitatory neurons were used to visualize excitatory neurons with two-photon imaging. For all hippocampal and some cortical imaging the Thy1-GCaMP6s GP4.3 line (RRID:IMSR_JAX:024275; Chen et al., 2013) was utilized. The remaining cortical imaging was conducted on mice resulting from a cross between the CAMK2a-tTa driver line (RRID: IMSR_JAX:007004) and a line expressing the calcium indicator GCaMP6s under the control of the tetracycline-responsive regulatory element (tetO, RRID:IMSR_JAX:024742; Wekselblatt et al., 2016). Mice were group-housed until the headplate implantation surgery (>P40), and housed individually after. The mice were maintained on a 12-hour light/dark cycle in the vivarium. Animals of either sex were selected for experiments. The animals were habituated to head fixation over a few days then trained to run and lick for hidden rewards in the visual VR in a series of steps that took 4–6 weeks. Mice were either water or food restricted to motivate behavior but given supplementary food or water to maintain 80 % of baseline weight.

### 3.2. Method details

#### 3.2.1. Surgical procedures

Mice underwent a headplate implantation and craniotomy in either the same or separate surgeries. First, the mice were implanted with custom designed metal headplates. In preparation, connective tissue was cleared from the surface of the skull and a thin layer of Vetbond was applied. Then the headplate was affixed, at an angle parallel to the site of imaging, with black dental acrylic (Lang Dental). The second procedure was a craniotomy. For cortical imaging, a 4 mm diameter cranial window was drilled using methods described previously (Salinas et al., 2017). The cranial window was centered either along the midline or 2 mm lateral to midline above the right hemisphere, 1.5 mm anterior to lambda. A 4 mm glass coverslip (World Precision Instruments) was placed over the exposed brain and sealed with Vetbond and black dental acrylic. Occasionally bone would grow underneath the coverslip, obscuring the field of view. An additional procedure would then follow to remove the current coverslip, delicately remove the bone growth and dura with a microscapel, and replace the coverslip. For hippocampal imaging, tissue over the somatosensory cortex was aspirated and replaced with a 1.6 mm cylindrical micro-optic plastic (MOP). MOPs were formed by curing the optical polymer BIO-133 (Han et al., 2021; Fort and Bardet, 2021) with 395 nm light in a custom-built aluminum mold. During all procedures, mice were anesthetized with isoflurane in O^2^ (2–3 % for induction, 1–1.5 % for maintenance). Carprofen (5 mg/kg, s.c.) and topical lidocaine (2 %, 20 mg/ml) were used as analgesics. Dexamethasone (4.8 mg/kg, i.m.) was administered 4 h before surgery to control inflammation. Sterile eye ointment (Rugby) was used to keep the eyes hydrated during the procedure. Body temperature was stabilized to 37° C with a heating pad under control of a rectal thermoprobe. The animals recovered on a warm heating pad post-surgery and were given daily injections of Carprofen (5 mg/kg, s.c.) for 3 days post-surgery.

#### 3.2.2. Visual virtual reality setup

The visual virtual reality (VR) system translated rotation of a 3D printed running wheel (37.7 cm circumference) into propulsion through a virtual circular track environment displayed on three tablets (T530NU Samsung). The animal was held by a head-fork over the wheel, and viewed the VR environment on tablets held at right angles 12 cm from the eyes (300° of visual field coverage along the azimuth). Rotations of the wheel were detected by a rotary encoder (Avago), processed by a data acquisition board (NIDAQ), and input into the computer. The animal’s licks in anticipation or during consumption of reward were detected by a capacitive lick sensor (Sparkfun), and also routed into the data acquisition board. A camera recorded the animal’s pupil (Allied Vision 1” GigE Vision). The data acquisition board output signals to open a solenoid valve for a specified amount of time, allowing water or diluted condensed milk to flow through to a reward spout placed in front of the animal’s mouth. Running speed, position along the track, licking, pupil size, and reward delivery were all recorded during the session.

Custom software written in MATLAB (Mathworks) managed the view of the VR environment. Based on movement of the wheel, the point of view along the circular track progressed forwards or backwards. The system updated at a 30 Hz refresh rate. Each VR environment was composed of a circular track with circumferences ranging from 314 to 502 cm. Distance in the real world was calibrated to match VR distance. Rewards were dispensed either automatically or in a lick-triggered manner at two locations within each environment (hidden reward sites). Each VR environment was also distinguished by floor, and wall images. Some environments had complex wall images (e.g. mountains) while others had simple repeating patterns. Visual objects consisted of 3D objects designed in Unity and positioned at various locations on either side of the circular track. The tunnel object was 30 cm long, while most other objects were 5–10 cm in width. Custom software (Smooth-Walk) was used to add these attributes, move the camera (mouse’s viewpoint), and wirelessly project the environments onto the tablets (Mao et al., 2020).

The animal began each session with the wheel blocked for 10–30 min. When unblocked, the animal ran for a short distance (126 cm) with the tablets blacked out, then entered into its first VR environment. The first VR environment the animal typically saw each session was the animal’s training (“familiar”) environment. The beginning of each lap was defined by the middle of the tunnel object. There were no inter-trial intervals as laps progressed continuously.

The VR environment could be programmatically changed. If the mouse was scheduled to transport into a new environment, the switch occurred when the animal was halfway through the tunnel. The animal therefore saw the environment instantly switch through the opening in the tunnel (all the tablets flashed briefly and then the new environment appeared). VR environment and object positions were recorded and saved at the end of every session.

#### 3.2.3. Environments

We designed eight visually distinct environments. Three of the environments (“Classroom, Landscape, and Sunset”), had complex backgrounds and were densely populated with objects on either side of the track. Each environment had a “track” that was 10 cm wide, which was visually distinct from the rest of the floor. The mouse’s position remained within the center of that track. Two versions of each environment were created, one “small” (314 cm in circumference) and one “large” (503 cm in circumference). No mouse saw both the small and the large versions of a single environment. Each environment had two different reward locations, and some were traversed in different directions (Landscape clockwise, i.e. the curve of the track appeared rightward, and Classroom and Sunset counter-clockwise, i.e. the curve of the track appeared leftward). Five additional environments (“Europa, Blue Room, Paw Room, Ornament Room, Dot Room”) contained distinctly patterned backgrounds (walls) and distinct floors (with a different pattern and color from the walls), and only eight objects each. The “track” on which the mouse would move within each environment was not distinguished from the rest of the floor, and was 377 cm in circumference. Each object was distinct, and located near (5–20 cm) the track. All environments except Europa had cylindrical walls on the inside and outside of the track (inside walls ~20 cm from the track; outside walls ~100 cm from the track), such that the mouse could not see objects on the opposite side of the track. (Europa only contained outer walls). Mice underwent training in one of the environments (most commonly the small version of Classroom), and the other environments were used for novel experiences. See Figs. 1 and 2, and supplementary videos for images of the environments. We did not analyze the differences in neural or behavioral responses to the different environments.

#### 3.2.4. Behavior

After recovering from surgery, mice were habituated to head fixation on top of the wheel for several days then taken through a 4–6 week training procedure familiarizing them to a single VR environment (“Familiar”). We developed a multi-stage training protocol for introducing mice to head-fixation, liquid reward (water or milk) delivery through a metal spout, introduction to running in VR, and transitioning from automatically delivered rewards to operant conditioning in which the mouse had to lick in the correct location (a 30–50 cm region), in order for a reward to be delivered. The last phase allowed us to have a behavioral read-out for how well the mouse understood the location of reward delivery.

Once the animal would regularly run over 10 laps and licked in anticipation of rewards, it was moved from the training setup to an identical VR setup underneath the microscope. On the imaging setup, mice were re-habituated to the familiar environment, then introduced on separate days to a series of novel environments. The novel environments were typically re-introduced for three days, either after 10–20 laps of the training (‘familiar’) environment or from the beginning of the new session. Mice were imaged on consecutive days with occasional breaks. Imaging on each mouse took anywhere from 1 to 8 weeks, so long as the quality of the cranial window was good and animals exhibited good behavior.

To quantify licking behavior, we first excluded any licks occurring up to 4 s after a reward delivery (see Suppl. Fig. S1A). Then we divided the track into 100 position bins, and determined if any of the remaining licks occurred in each bin on each lap (this ensured that bursts of licks were not weighted more than single exploratory licks). The ten bins surrounding each reward site were classified as correct locations, and the rest of the track contained non-specific licking. This allowed us to analyze the behavior in the same way regardless if the rewards were delivered automatically (as was the case in most novel-environment laps), or if the mouse was required to lick (within a 10 bin zone surrounding at the auto-deliver location) to trigger the reward delivery (most familiar-environment laps). Lick precision was calculated as the ratio of the anticipatory lick-bins to the difference between total lick-bins and post-reward lick-bins. The chance level for this calculation is 0.2 (10 anticipatory lick-bins for each of 2 rewards divided by 100 total bins). For lap-wise analyses, any laps in which no licks occurred were assigned a precision score of 0, and so in those analyses, some laps could score below 0.2.

#### 3.2.5. Two photon imaging

Calcium transients from GCaMP6s expressing excitatory cells were recorded using a two-photon mesoscope (Neurolabware). Excitation from a laser tuned to 920 nm (Insight X3, SpectraPhysics) was phase modulated by a pockels cell (Conoptics) then guided through table optics to a water immersion 10 mm objective (numerical aperture 0.5). Brain regions were imaged through this objective by scanning the laser bidirectionally across a specified field of view using resonant and galvanometer mirrors (Cambridge Technology) and an electrically tunable lens (Optotune). Emissions were captured and amplified by GaAsP PMT and filtered using a 510/84 nm BrightLine bandpass filter (Semrock).

The objective was lowered to focus on a depth of view between 100 and 300μm below the pia in cortex, and 200–300 μm below the alveus in hippocampus. For retrosplenial imaging, one (unilateral) or two (bilateral) regions of interest (ROIs) were specified for fast imaging (each typically 1000 μm x 600 μm) from an initial 4 mm large panoramic field of view of the posterior cortex. Laterally, these ROIs were placed as close as possible to the central sinus, at maximum 1.5 mm lateral, as measured by the width of the ROI windows. Meanwhile, cells across the bottom 4 mm of posterior cortex were sampled across mice while avoiding regions with bone growth or excessive vasculature. We felt confident ROIs within this area would remain within RSC, given that the craniotomy was stereotactically positioned over posterior cortex and RSC extends extensively up the rostral-caudal length of the mouse neocortex. Indeed, separate experiments (not reported here) used wide-field calcium imaging to map the locations of visual and somatosensory cortices in a subset of the mice also used in this study. These experiments verified our ROIs placements for RSC recordings were more medial to visual and posterior and medial to somatosensory cortices. Hippocampal CA1 ROIs were placed based on optimal appearance of cell bodies within the limited constraints of the stereotactically placed MOPs. Typically, two 850 μm x 530 μm ROIs were placed adjacent to one another and the electrically tunable lens was used to switch depths between ROIs to accommodate for the curvature of the CA1 layer. ROIs were recorded at a frame rate of 6–8 Hz using the Scanbox acquisition software (Neurolabware).

### 3.3. Quantification and statistical analysis

In the text, ‘+ /−’ always indicates standard deviation. Parametric statistics were used for hypothesis testing. Linear mixed effects models were used wherever possible, to account for the hierarchical structure of the data. Signals coming from the same sessions or animals, which may influence the results but are not of importance to the experiment, were set as random effects in the models. In figures, error bars indicate standard error of the mean. Analyses were conducted with MATLAB, Python 3 (primarily using the numpy and pandas packages for data manipulation; statsmodels, scipy, pingouin, and rpy2 for stats; and seaborn and statannotations for plotting), and GraphPad Prism.

#### 3.3.1. Pre-processing

The imaging data was converted into the TIFF file format using custom software, and then run through the python Suite2P pipeline for registration and segmentation (Pachitariu et al., 2017). Trained undergraduate technicians manually curated cells based on morphology of the soma and plausibility of activity traces. The time varying fluorescence for each of the curated cells was taken as the average fluorescence of all pixels in each cell mask. These fluorescence traces then entered a MATLAB analysis pipeline. First, the fluorescence of each cell body was normalized by the 10 pixel wide surrounding neuropil signal (F(*t*) = F_soma_(*t*) - 0.7*F_neuropil_(*t*); Chen et al., 2013). Next, the relative fluorescence change (ΔF/F_0_) was calculated as follows: The running baseline (F_0_(*t*)) was calculated for each time *t*, by smoothening the fluorescence trace based on a running average over a time window *t_1_*, and taking the minimum value from the smoothened trace within a time window *t_2_* behind the current time point, *t*. *t_1_* was set to 5 s and *t_2_* was set to 60 s (Danielson et al., 2016). Deconvolved spikes output from Suite2P was used for certain analyses as an alternative to the ΔF/F. Suite2P utilized the well-validated OASIS algorithm (Friedrich et al., 2017), which optimized correlation between spikes inferred from two-photon fluorescence from calcium indicators with simultaneously recorded real spikes (for instance, from the GENIE dataset). As we did not have ground truth electrophysiology with our recordings from this project, we cannot determine the false positives or false negatives, but felt confident the technique was appropriate based on testing on GCaMP6s data in the original OASIS algorithm paper and visual inspection of the deconvolution results. The OASIS algorithm produces the time bin (image frame) of each putative spike, as well as the amplitude of the spike. Noise in the fluorescence signal results in the identification of many very small amplitude spikes. For place field size and center of mass calculations, these low amplitude spikes were removed by implementing a threshold to remove the 50 % lowest amplitude spikes. For spatial information and decoder error calculations, the amplitudes of all spikes (including low amplitude spikes) occurring in each position bin were averaged to generate a spatial “firing rate” map for each lap. Then, this map was smoothed with a hanning window of 2 spatial bins (6–10 cm).

#### 3.3.2. Criteria for inclusion of data

There was a lot of variability between mice in licking behavior, training required to get to the imaging stage, and number of laps run per session, and so to ensure quality data, we removed datasets in which the mouse ran less than 10 laps in a single environment, we were not able to detect 50 or more cells, or the decoder error for the familiar environment was greater than 30 cm on the day prior to novel environment introduction. In most cases, if mice did not reach the decoder error criterion within one week of imaging in the familiar environment, we stopped collecting data from that mouse, but in some cases the data was removed post-hoc.

#### 3.3.3. Position binning

Certain signals, such as velocity and cell activity, were position binned and occupancy normalized. All signals were resampled to the slowest signal, which was the imaging rate (6–8 frames/s). First, any time bins in which the velocity was less than 1 cm/s were eliminated, so that we did not analyze periods when the mouse was stopped or running backwards. Then we constructed an M x N matrix, where M is the total number of laps the animal ran in the environment, and N is the number of bins into which the circumference of the circular track was divided (100). The average signal at each bin was then calculated by summing over the time period the animal spent at that bin and dividing by the length of time spent at that bin. This resulted in a rate of activity for each entry of the position binned matrix. The bin size was 3–5 cm, depending on the size of the environment. When the mouse ran faster than the imaging rate/bin (18–40 cm/s, depending on the environment size and imaging rate), some bins would register an occupancy of 0, and thus would be stored as NaN. This happened rarely for most mice, and since most analyses involved averaging across laps or bins, those bins would just be excluded. Note that the first and last bins of each lap are neighboring positions on the track, and are both inside the tunnel of each environment.

#### 3.3.4. Visualization of population activity

To visualize position correlated sequences, lap-averaged activity of all recorded (or just position-correlated) cells were plotted as a function of bins in the environment. Cells were ordered along the y-axis by the position of their most active bin (i.e. cells ordered based on how early along the track activity peaked). To ensure that any observed position correlated sequences were due to the cells consistently firing at the same positions, a different set of laps were averaged when determining the peak bin (a random subset of 5 of the final 10 laps) and for plotting (the rest of the laps in that environment). To see if the same sequence persisted across both environments A and B, the activity in environment B was plotted by sorting cells by their peak bin in A, and vice versa.

#### 3.3.5. Classifying position-correlated cells (PCCs)

To identify cells that were position-correlated in a particular environment, we used a three-step criterion. First, the position-binned fluorescence activity (ΔF/F) of each cell was averaged across laps, smoothed with a Hanning window of 5 bins, and the local peaks of this average trace were found. Any peaks that were higher than 3.5 times the 50th percentile of this trace were considered candidate place fields. The boundaries of these candidate place fields were set at the closest position at which the activity dropped below the 50th percentile, or the closest trough after which the trace reached below 70 % of the peak (whichever came first). Second, the peak activity on each lap within the boundary of each field was compared to the baseline activity (50th percentile minus 5th percentile of activity in all bins and all laps). The in-field activity had to be 3.5x greater than the baseline in at least ⅓ of laps, or 5 laps (whichever was greater), to continue considering it as a place field. Finally, the size of the field was calculated based on the previously set boundaries, and any fields less than 20 cm, or greater than 150 cm were eliminated. The field size criterion eliminated less than 2 % of all fields in each dataset. Cells were considered position-correlated if they were determined to have at least one place field. For some analyses, place fields were considered independently of the cell they belonged to, so that multiple fields belonging to one cell could be analyzed.

The thresholds in this criterion (50th percentile and 3.5x greater peak than baseline) had been determined by an experimenter visually inspecting resulting place fields for two hippocampal and two retrosplenial datasets. After this, the criteria were set the same for all datasets regardless of brain region. These thresholds are somewhat arbitrary, and not very stringent. Therefore, we considered all cells without using this criterion wherever possible (e.g. spatial information calculations, in-out activity ratios etc.). The percent of cells that pass the criterion in each dataset may reflect a combination of many factors, including the familiarity of the animal with the environment, the brain region, the size of the environment, imaging quality, and the number of environments visited. The last two are factors, because cells that do not have place fields do not fire often (especially in the hippocampus), and thus may be missed by our cell detection algorithm, especially when the images are dim, or not much imaging outside of the behavior period is considered. We attempted to limit this impact by imaging for 5–30 min before and after each running session (while the animal was resting on the immobile wheel in the dark), and using the whole session for cell detection.

#### 3.3.6. Place field size and center of mass (COM) calculation

After identification of each place field and its boundaries (see Classifying PCCs), activity and deconvolved calcium events (DCEs) within those boundaries were identified for each lap. Only the activity within these boundaries was used to analyze that field, so that cells with multiple place fields could be analyzed with each field considered separately. We used two methods to calculate field size and COM. The first method used calcium activity (ΔF/F). To calculate place field size, we found the peak activity within place field boundaries in each lap, and then counted the number of bins with activity at least half of that peak. This method was designed to be analogous to the “width at half max.” Place field COM was calculated by weighting the position of each bin with the ΔF/F activity in that bin and averaging. For each field, the COM on each lap was subtracted from the average COM across laps. Any laps with peak activity below the 3.5*baseline activity threshold (see Classifying PCCs) were excluded for that field. This ensured that place fields were only analyzed after their formation.

We also used DCEs to calculate place field size and center of mass. To remove noise, we used only the 50 % of DCEs with the highest amplitudes (see Pre-Processing). Only laps with 2 or more remaining DCEs were used for each field. Field size was calculated as the distance between the first DCE and the last DCE in each lap. Place field COM was calculated by weighting the position of each bin with the summed DCE (occupancy normalized) amplitude in that bin and averaging. The COM on each lap was subtracted from the average COM across laps.

#### 3.3.7. Population vector correlations

The population vector is a list of the activities of all simultaneously imaged cells in a particular time or position bin. To compare activity between laps and between environments, we calculated the activity of all cells in each position bin in a single lap, or averaged across three consecutive laps. Then we correlated the population vector in each position bin, with the same in each position bin in a different set of laps. This resulted in a matrix of correlations (Fig. 3A&B). The correlations along the diagonal represent the same position bins correlated between lap intervals. We averaged the correlations along the diagonal to get the average correlation of one lap interval with another lap interval, either in the same or in different environments. Because some environments were different sizes, we divided all environments into 100 bins, differing in size (from 3 to 5 cm) between environments, in order to have square population vector correlation matrices. This means that moving along the diagonal does not correspond to the same distance in different environments. However, if there was a correlation between environments at corresponding distances (instead of corresponding bins), this would be seen as an increased correlation along y = 3/5x (for example), instead of the diagonal, which was not observed.

#### 3.3.8. Spatial information

Spatial information content (SI) quantifies the information available to locate the animal based on neuronal activity (Skaggs et al., 1992). We calculated the SI for each cell based on the lap-averaged deconvolved signal based on this formula:

$$SI=\overset{N}{\underset{i=1}{\sum }}p_{i}\frac{f_{i}}{f}\log_{2}\frac{f_{i}}{f}$$

N stands for the total number of bins (100), p_i_ is the probability of occupying the i^th^ bin, f_i_ is the deconvolved activity in the i^th^ bin, and f is the activity averaged across all f_i_ bins. The measure is quantified in bits per deconvolved calcium event. The deconvolved activity was shuffled 100 times (each lap was circularly shifted by a random number of bins) to also obtain a null distribution of spatial information scores.

#### 3.3.9. Z-scored cell activity by lap

To examine how neural activity changed across laps, the position binned activity of each cell was first lap-averaged then z-scored against the mean and standard deviation of the lap-averaged activity of all cells and all laps in the session (in order to eliminate any differences in fluorescence levels between animals, but not between cells in the same mouse). The cells were split based on whether fields were in the familiar, novel, or both environments. For cells of each category, the activity was plotted as heat maps with cells (combined across RSC or CA1 sessions) sorted along the y-axis by z-scored activity of the final five laps in the novel environment (Fig. 4A). To determine the average trends across laps, activity in the heat maps were averaged first by cells from the same session, then by sessions from the same animal, and finally across sessions. This produced line graphs of activity as a function of lap number for cells in each category, for CA1 and RSC (Fig. 4B). The thin lines represent standard error.

The change in activity across the transition between environments was quantified by taking each cell’s z-scored activity averaged from the first three laps of the novel environment, and comparing it to the average activity in the last three laps of the familiar environment. Again the cells were separated by whether their fields were in just the familiar, novel, or both environments, then the activity of cells in each category were averaged by session, then between sessions from the same animal, and finally across sessions. Sidák’s multiple comparisons test was used to determine which categories of cells had significantly different activity between familiar and novel environments.

#### 3.3.10. Bayesian decoding

A Bayesian decoder was used to estimate the animal’s position based on deconvolved neural population activity (Zhang et al., 1998). At every time point (t), the posterior probability (p) of being at a particular position (x) on the track, given the neural population activity (n_t_), was calculated based on the prior probability of being at a particular position multiplied by the likelihood of this neural activity being produced at that position, divided by a normalization term (Bayes’ theorem):

$$p\left(n_{t}\right)=p(x)*p(x)/p\left(n_{t}\right)$$

The prior probability (p(x)) is the relative occupancy of each bin. The probability of neural activity at a given position is derived from the average deconvolved population activity across position bins, smoothed with a Hanning window of 9 bins. Both of these probabilities are calculated with leave one out cross-validation; such that time points corresponding to when the animal is on a particular lap are decoded based on average activity across all other laps besides the current lap. n_t_ was calculated as the mean activity within a time window of 2 s, calculated at each image frame (125 or 167 ms intervals). To best compare across datasets, we used a random subset of 146 neurons (the minimum recorded from all but 1 mouse, which was excluded from this analysis). The decoded position was defined as the position with the highest probability at each time bin, and the absolute value of the difference between true position and decoded position was defined as the decoder error. Random selection of a subset of neurons was repeated 100 times, and the mean decoder error was averaged across those trials. We also computed decoder error using subsampling to 50, 100, 200, 300, and 500 neurons, to determine the effect of this parameter (see Suppl. Fig. S4B). Error decreased proportionally with number of neurons across datasets.

## Supplementary Material

supplementary text and figures

supplementary video 1

supplementary video 3

supplementary video 4

supplementary video 2

## Figures and Tables

**Table 1:** Summary of all statistics tests.

Figure	Variable	N	Test	Null hypothesis	Results	Statistic
2D	Location of field in familiar vs. location in novel	RSC: 689 fields RSC: 436 fields not near tunnel	Correlation Correlation	Locations are uncorrelated across environments Locations of fields away from the tunnel are uncorrelated	Reject null Reject null	r = 0.28; p < 0.001 r = 0.14; p = 0.003
2E	Percent of RSC cells with fields in both environments	RSC: 18 sessions	Linear Mixed Effects Model	Percent of cells is same as mathematically expected value Percent of cells away from tunnel is same as expected value	2.41 % higher than expected; reject null 1.77 % higher than expected; reject null	p < 0.0001 p < 0.0001
2G	Location of field in familiar vs. location in novel	CA1: 543 fields	Correlation	Locations are uncorrelated across environments	Accept null	r = 0.08; p = 0.06
2H	Percent of CA1 cells with fields in both environments	CA1: 10 sessions	Linear Mixed Effects Model	Percent of cells is same as mathematically expected value Percent of cells away from tunnel is same as expected value	0.72 % higher than expected; accept null 0.53 % higher than expected; accept null	p = 0.103 p = 0.191
3C	PV correlation with last 3 laps in familiar	RSC: 18 sessions CA1: 10 sessions 18 vs. 10 sessions	One way repeated measures ANOVA One way repeated measures ANOVA Two way repeated measures ANOVA	There is no difference across laps There is no difference across laps There is no interaction between lap and brain region	Reject Null Reject Null Accept Null	F= 146; p < 0.0001 F= 54; p < 0.0001 F= 0.55; p = 0.47
3D	PV correlation with late laps in novel	RSC: 18*10 laps CA1: 10*10 laps 180 vs. 100	Linear regression Compare linear regression parameters	There is no relationship between PV correlation and lap RSC and CA1 slopes are equal RSC and CA1 intercepts are equal	R^2^ = 0.15 R^2^ = 0.088 Accept null Reject null	p < 0.0001 p = 0.0028 F(1276)= 0.44; p = 0.51 F(1277)= 83; p < 0.0001
3E	PV correlation with 7 laps away	RSC: 18*6 laps CA1: 10*6 laps	Linear regression	There is no relationship between PV correlation and lap	R^2^ = 0.047 R^2^ = 0.026	p < 0.024 p = 0.22
		108 vs. 60	Compare linear regression parameters	RSC and CA1 slopes are equal RSC and CA1 intercepts are equal	Accept null Reject null	F(1164)= 0.015; p = 0.90 F(1165)= 64; p < 0.0001
S4D	Mean decoder error	RSC: 15 mice; CA1: 6 mice	Two-way repeated measures ANOVA	Familiar and novel environments are the same RSC and CA1 are the same	Reject null Accept null	F= 40.8; p < 0.0001 F= 0.092; p = 0.51
Not shown	Decoder error in best 5 laps	RSC: 15 mice; CA1: 6 mice	Two-way repeated measures ANOVA	Familiar and novel environments are the same RSC and hippocampus are the same	Reject null Accept null	F= 16.5; p = 0.0007 F= 0.67; p = 0.42
3F	Decoder error in each novel lap	RSC: 17*15 laps CA1: 10*15 laps 255 vs. 150	Linear regression Fit an exponential decay with offset	Slope is not different from zero Line is a better fit than exponential One curve fits both datasets	R^2^ = 0.14 R^2^ = 0.16 Reject null; Accept null; best fit curve: Y= (88–39)*e^−0.29X^ + 39	F= 41; p < 0.0001 F= 28; p = 0.0002 RSC: F= 15; p < 0.0001 CA1: F= 5.8; p = 0.017 F(3399)= 2.1; p = 0.11
3G	Lick precision in each novel lap	RSC: 18*15 laps CA1: 10*15 laps	Two-way repeated measures ANOVA	There is no effect of laps There is no difference between RSC and CA1 mice	Reject null Accept null	F= 2.4; p = 0.003 F= 0.055; p = 0.46
4B	Activity in last 3 laps of familiar vs. first 3 laps of novel	RSC: 18 datasets CA1: 6 mice	Sidak’s multiple comparisons test Sidak’s multiple comparisons test	The activity of PCCs in each category doesn’t change across the environment transition	Familiar only PCCs: reject null PCCs in both: reject null Novel only PCCs: accept null Familiar only PCCs: reject null PCCs in both: reject null Novel only PCCs: reject null	t = 5.4; p < 0.0001 t = 3.1; p = 0.012 t = 1.8; p = 0.27 t = 5.0; p < 0.0001 t = 3.2; p = 0.013 t = 4.1; p = 0.0009
4C	Mean activity of all cells in familiar laps Mean activity of all cells in novel laps	RSC: 18 datasets CA1: 10 datasets RSC: 18 datasets CA1: 10 datasets	Linear regression Linear regression	Mean activity doesn’t change across laps Mean activity doesn’t change across laps	Accept null Accept null Accept null Reject null	F= 2.5; p = 0.12 F= 2.5; p = 0.12 F= 1.9; p = 0.17 F= 5.5; p = 0.021
4E	In-out activity ratio in novel laps	RSC: 18*15 laps CA1: 10*15 laps	Linear regression	Slope is not different from zero	Reject null, R^2^ = 0.18 Reject null, R^2^ = 0.11	F(1268)= 59; p < 0.0001 F(1148)= 19; p < 0.0001
		270 vs. 150	Compare linear regression parameters	RSC and CA1 slopes are equal RSC and CA1 intercepts are equal	Accept null Accept null	F(1416)= 1.4; p = 0.24 F(1417)= 0.23; p = 0.63
	In-out activity ratio in familiar laps	RSC: 18*15 laps CA1: 10*15 laps	Linear regression	Slope is not different from zero	Reject null, R^2^ = 0.029 Accept null	F(1178)= 5.2; p = 0.023 F(1,95)= 0.0014; p = 0.97
S5B	Mean activity at different running speeds	RSC: 18* up to 20 bins CA1: 10* up to 20 bins	Linear regression	Slope is not different from zero	Reject null, R^2^ = 0.093 Reject null, R^2^ = 0.56	F(1262)= 27; p < 0.0001 F(1153)= 196; p < 0.0001
		264 vs. 155	Compare linear regression parameters	RSC and CA1 slopes are equal	Reject null	F(1415)= 23; p < 0.0001
S5C	Running speed in novel laps	RSC: 18*15 laps CA1: 10*15 laps	Two-way repeated measures ANOVA	There is no effect of laps There is no effect of brain region	Reject null Accept null	F= 4.3; p < 0.0001 F= 0.48; p = 0.50
		28 sessions	Tukey’s multiple comparisons test	Identify which laps show a difference	Lap 1 is different from all other laps; all other lap by lap comparisons are not significant	p < 0.01 or lower
5C	COM shift in familiar laps	RSC: 18 *10 laps CA1: 10 * 10 laps	Linear regression	Slope is not different from zero	Reject null: Y= −0.17X+ 1.4 R^2^= 0.11 Reject null: Y= −0.65X+ 4.7 R^2^= 0.51	F= 22; p < 0.0001 F= 103; p < 0.0001
		180 vs. 100 CA1: 10 *10 laps	Compare linear regression parameters Fit an exponential decay with offset	RSC and CA1 slopes are equal Line is a better fit than exponential	Reject null Reject null: Best fit curve: Y= (37.4 +1.5) *e^−0.28 X^ - 1.5	F= (1276)= 51; p < 0.0001 F= 12; p = 0.0008
	COM shift in novel laps	RSC: 18 * 15 laps CA1: 10 * 15 laps	Linear regression	Slope is not different from zero	Accept null Reject null: Y= −0.28X+ 3.5 R^2^= 0.22	F= 2.5; p = 0.12 F= 42; p < 0.0001
		270 vs. 150 CA1: 10*15 laps	Compare linear regression parameters Fit an exponential decay with offset	RSC and CA1 slopes are equal Line is a better fit than exponential	Reject null Reject null: Best fit curve: Y= (5.2 +0.32) *e^−0.19X^ – 0.32)	F= 22, p < 0.0001 F= 5.8; p = 0.017
5D	Place fields with significant COM shift	RSC: 16 mice CA1: 6 mice	Two-way repeated measures ANOVA	The percent of place fields with significant backward shift is the same in CA1 and RSC The percent of place fields with significant backward shift is the same in the familiar and novel environments	Reject null Accept null	F(20,1)= 47; p < 0.0001 F(20,1)= 0.40; p = 0.53
5E	Mean place field size	RSC: 16 mice CA1: 6 mice	Two-way repeated measures ANOVA	Field sizes are the same in novel and familiar RSC and CA1 have the same field sizes	Accept null Reject null	F(20,1)= 0.060; p = 0.81 F(20,1)= 6.2; p = 0.022
5F	Place field size in familiar laps	RSC: 18 * 10 laps CA1: 10 * 10 laps	Linear regression	Slope is not different from zero	Reject null: Y= −0.0074X+ 1.0 R^2^= 0.086 Accept null	F= 17; p < 0.0001 F= 0.72; p = 0.40
		180 vs. 100	Compare linear regression parameters	RSC and CA1 slopes are equal	Accept null	F(276,1)= 2.8; p = 0.098
	Place field size in novel laps	RSC: 18 * 15 laps CA1: 10 * 15 laps	Linear regression	Slope is not different from zero	Reject null Y= 0.0029X+ 0.98 R^2^= 0.023 Reject null: Y= −0.0060X+ 1.05 R^2^= 0.085	F= 6.3; p = 0.013 F= 14; p = 0.0003
		270 vs. 150	Compare linear regression parameters	RSC and CA1 slopes are equal	Reject null	F= 20; p < 0.0001
6A	Lick precision on each day in novel	RSC: 18 datasets * 3 days CA1: 10 datasets * 3 days	Mixed effects analysis	Lick precision is the same across days Lick precision is the same in RSC and CA1 mice	Reject null Accept null	F(2,47)= 9.9; p = 0.0002 F(l,26)= 2.6; p = 0.12
6D	Pupil size across days	RSC: 16 datasets * 2 days CA1: 10 datasets * 2 days	Mixed effects analysis	Pupil size is the same on day 1 and day 3 Pupil size is the same in RSC and CA1 mice	Reject null Accept null	F(1,19)= 11; p = 0.0032 F(1,24)= 0.056; p = 0.82
6E	Pupil size across laps in novel day 1	RSC: 16 datasets * 15 laps CA1: 10 datasets * 15 laps	Two-way repeated measures ANOVA	Pupil size doesn’t change across laps Pupil size is the same in RSC and CA1 mice	Reject null Accept null	F= 6.1; p = 0.0042 F= 0.015; p = 0.90

## Appendix A. Supporting information

Supplementary data associated with this article can be found in the online version at doi:10.1016/j.pneurobio.2025.102867.
