# Supplementary material for: Place field dynamics in retrosplenial cortex compared to hippocampus
Source: Prog Neurobiol. Author manuscript; Available in PMC 2026 Apr 19. (PMC13092332; doi:10.1016/j.pneurobio.2025.102867)
Supplement: supplementary text and figures [file NIHMS2158806-supplement-supplementary_text_and_figures.docx]

**Table S1: Datasets used in novel environment analyses.**

| Mouse | Date | Familiar Environment | Novel Environment | Area | Hemi-sphere | Depth (um) | Sex | Genotype | Age (days) |
| --- | --- | --- | --- | --- | --- | --- | --- | --- | --- |
| AZ2 | 6-30 | Sunset | Classroom Large | RSC | R | 155 | F | Thy-1 | 303 |
| AZ2 | 7-12 | Sunset | Dot Room | RSC | R | 142 | F | Thy-1 | 315 |
| AZ2 | 8-9 | Sunset | Paw Room | RSC | R | 150 | F | Thy-1 | 343 |
| DB10 | 3-9 | Classroom | Paw Room | RSC | R | 187 | F | Niell | 145 |
| DB12 | 3-18 | Classroom | Ornament Room | RSC | R | 162 | F | Niell | 154 |
| DB13 | 3-30 | Classroom | Ornament Room | RSC | R | 494 | M | Niell | 166 |
| DB15 | 5-24 | Classroom | Paw Room | RSC | R | 152 | M | Niell | 192 |
| DB19 | 10-5 | Classroom | Sunset Large | RSC | L | 100 | M | Niell | 204 |
| DB26 | 1-5 | Classroom | Dot Room | RSC | R | 170 | M | Niell | 117 |
| DB27 | 1-24 | Classroom | Blue Room | RSC | R | 289 | F | Niell | 120 |
| ZD32 | 6-12 | Landscape | Classroom | RSC | L | 210, 300 | M | Niell | 258 |
| ZD40 | 8-17 | Classroom | Europa | RSC | R | 250, 177 | M | Thy-1 | 230 |
| ZD46 | 3-18 | Europa | Classroom | RSC | R | 157 | F | Thy-1 | 297 |
| ZD55* | 5-9 | Classroom | Europa | RSC | R | 71 | F | Niell | 219 |
| ZD62 | 4-23 | Europa | Landscape | RSC | L,R | 236, 189 | M | Niell | 152 |
| ZD63 | 6-3 | Classroom | Landscape Small | RSC | L,R | 174, 136 | F | Niell | 210 |
| ZD66 | 5-28 | Classroom | Large Europa | RSC | L,R | 234, 157 | F | Niell | 173 |
| ZD69 | 9-17 | Europa | Classroom | RSC | R | 209, 429 | M | Niell | 309 |
| MOP14 | 4-15 | Classroom | Paw Room | CA1 | L |  | F | Thy-1 | 192 |
| MOP16 | 5-28 | Classroom | Sunset Large | CA1 | L |  | M | Thy-1 | 235 |
| MOP17 | 8-5 | Classroom | Paw Room | CA1 | L |  | M | Thy-1 | 304 |
| MOP17* | 9-1 | Classroom | Sunset Large | CA1 | L |  | M | Thy-1 | 331 |
| MOP17** | 9-6 | Classroom | Landscape | CA1 | L |  | M | Thy-1 | 336 |
| MOP18 | 10-4 | Classroom | Ornament Room | CA1 | L |  | M | Thy-1 | 199 |
| MOP18 | 10-18 | Classroom | Dot Room | CA1 | L |  | M | Thy-1 | 213 |
| MOP22 | 4-6 | Classroom | Sunset Large | CA1 | L |  | M | Thy-1 | 218 |
| MOP22 | 4-19 | Classroom | Dot Room | CA1 | L |  | M | Thy-1 | 231 |
| MOP23 | 7-20 | Classroom | Dot Room | CA1 | L |  | M | Thy-1 | 323 |

Information about all datasets is displayed. All mice were trained in a “familiar” environment for 3 weeks or more, and then introduced to a novel environment during imaging. On the first day in the novel environment, the mouse ran for ~10 min (10-20 laps) in the familiar environment and then was “teleported” into the novel scene. Most mice ran in each novel environment for three days. One asterisk indicates datasets that did not include a day 3, and two asterisks indicate a dataset without days 2 or 3. In some cases, the same mouse was introduced to additional novel environments (with several days intervening between the last exposure to the first and the first exposure to the additional novel environment), and those sessions were also included.

We used a different genotype to study RSC and CA1 (but both were on a B6 background), because of different GCaMP6s expression levels of each genotype. The Thy1-GCaMP6s (GP4.3) genotype has high expression in hippocampus, and moderate in cortex (Dana et al., 2014). Both are expressed mainly in pyramidal cells, but the Niell genotype is highly specific for mature glutamatergic neurons (Wekselblatt et al., 2016), while Thy1 may show some expression in a small percentage of glial cells, and shows some variability in expression levels between cortical layers and regions. To test if genotype differences could account for some of the difference between CA1 and RSC, we used Thy1 mice for some RSC implants. We did find that Thy1 mice had somewhat smaller RSC place field sizes (29 cm vs 38 cm, T-test: p=0.038), and expressed a higher number of place fields per meter (p=0.0023 in familiar and p=0.0034 in novel environments). There was no difference between Thy1 RSC mice and Thy1 CA1 mice in these measures, and therefore the difference between CA1 and RSC in number of PCCs can be at least somewhat explained by the genotype differences. However, there were no differences between genotypes in decoder error, behavior (lick precision), or percent of place fields showing backwards shift. A larger number of CA1 cells showed significant backwards shift compared to RSC cells recorded in Thy1 mice. Thus, all major findings hold up regardless of genotype.

In addition, some mice were implanted with cranial windows across the midline, so we could simultaneously image the right and left RSC, while other mice were implanted unilaterally to study other brain regions (not shown). We compared place field sizes in right and left RSC, taking into account the direction of running in each environment (because clockwise running was associated with faster optic flow in the right visual field, which is projected to the left hemisphere, whereas this is reversed in counterclockwise running), and did not find any significant differences.

**Figure S1: Behavioral measures.**


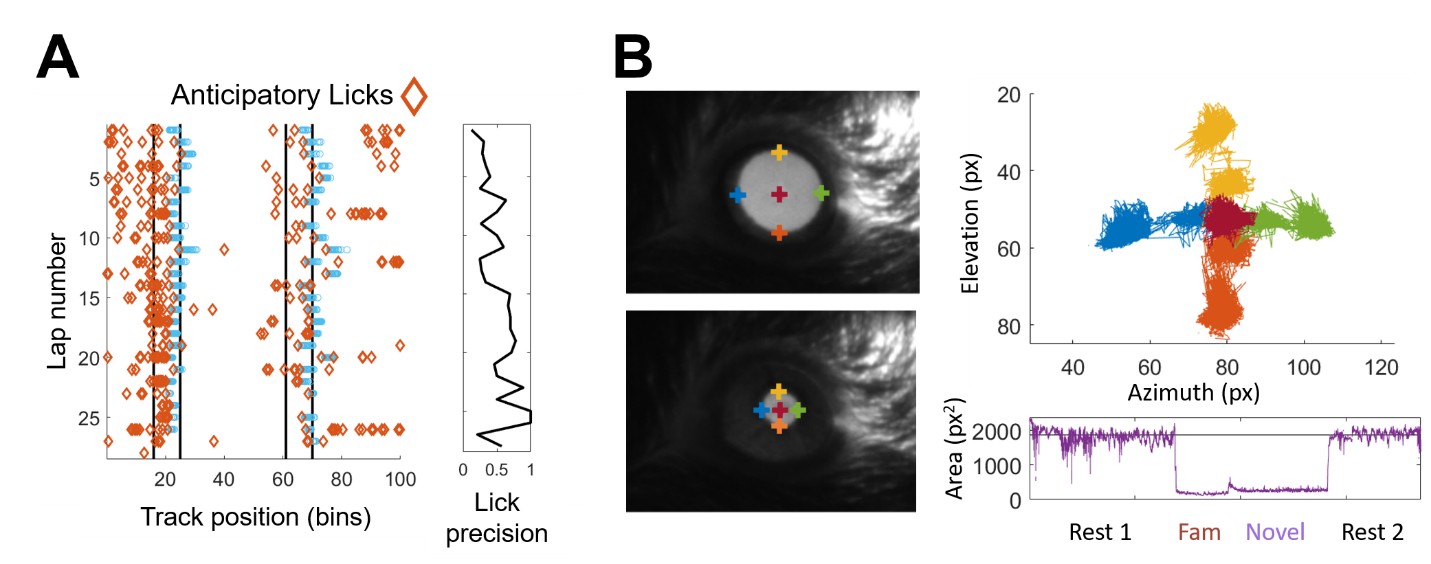


We used a touch sensor on the lick spout for reward delivery, and a camera focused on the eye or face to monitor mouse behavior as they explored the novel environments. **A.** Mice were encouraged to lick the reward spout in anticipation of reward locations, by making reward delivery contingent on active licking in two specific zones of each environment. Mouse behavior was evaluated by calculating the proportion of anticipatory licks in the reward zones. In this example from one mouse in a novel environment, light blue circles indicate consummatory licks, which occurred within four seconds after a reward was delivered. All other licks are considered anticipatory licks (orange diamonds). Reward zones are indicated by black boundaries, and the proportion of anticipatory licks in these zones was calculated for each lap and plotted on the right. Each reward zone was 1/10^th^ of the track length, and thus licking at random would result in a lick proportion of 0.2. **B**. In order to measure the attention mice were paying to the environments, we identified the pupil in videos of the mice using DeepLabCut. The center, top, bottom, left and right edges of the pupil were marked in each movie frame. Top left image shows a video frame in which the mouse is resting in the dark with tablets turned off. The bottom left shows a frame from a run session in which the VR is displayed. On the top right, data points from all image frames collected during a session are plotted in x-y coordinates. Pupil area was calculated as an oval with radii marked by the top and bottom, and left and right points. On the bottom right, the area of the pupil is plotted across time for the example session, including two rest periods, and running in the familiar followed by the novel environment. Pupil area was normalized by the average size during the rest periods in each session (black horizontal line).

**Figure S2: Probability of place field allocation.**


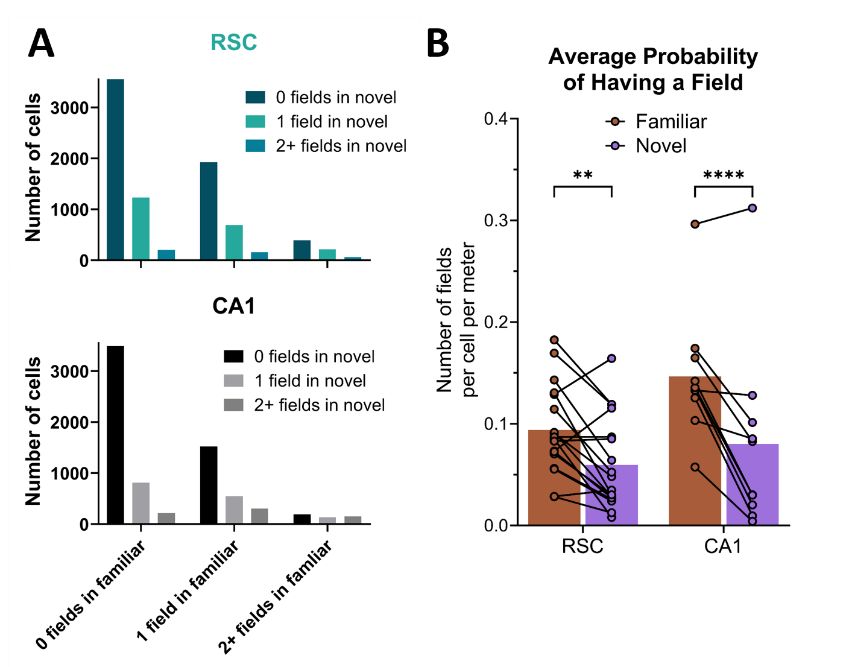


In figure 2, we showed that the proportion of cells with fields in both environments is the same (in CA1) and almost the same (in RSC) as that predicted by random allocation with replacement (RAR). This assumption is incorrect in the hippocampus (Witharana et al., 2016), likely because pyramidal cells show something similar to a log normal distribution in propensity to fire (Rich et al., 2014; Lee et al., 2020). **A.)** Our data are consistent with the latter studies, because we also found that fields with one or more fields in one environment were more likely (than cells with no fields) to have one or more fields in the other environment (Fisher’s exact test; RSC: Χ^2^=256, df=1, p<0.0001; CA1: Χ^2^=156, df=1, p<0.0001). This was even more true for cells with two or more fields in one environment (Chi square test; RSC: Χ^2^=363, df=4, p<0.0001; CA1: Χ^2^=545, df=4, p<0.0001). This may have partially been due to differences between mice (e.g. exact imaging location), but on the individual mouse level, 14/18 RSC recordings and 3/10 CA1 recordings showed a statistically significant disproportionate allocation of fields (Chi square test, X^2^<3.841, df=1, p<0.05). However, these differences in propensity to fire were not large enough to significantly impact the proportion of cells in both environments (Fig. 2H), because only about half the cells had any fields in either environment, and very few had 2 or more fields in a single environment. The propensity of a cell to fire predicts how many fields a cell will have in a large environment, but not where those fields will be. Thus, in our relatively small environments compared to Lee et al., 2000, there was a low correlation between number of fields in familiar vs. novel (RSC: R=0.17, p<0.0001; CA1 R=0.24, p<0.0001), and thus a very low ability to predict if a cell was going to fire in the novel environment. **B.)** Witharana et al. used smaller environments, but their analysis was a bit different than ours. They used the proportion of cells active in one familiar environment to predict the proportion of cells active in X (familiar and novel) environments, based on the RAR assumption, and found this assumption largely overestimated the total number of active cells. Instead, we used the proportion of cells active in both the familiar and novel environments to predict the overlap. This may account for some of the difference in our results, since the number of fields per cell per meter is significantly lower in the novel than that in the familiar environment (63% of familiar in RSC, Sidak’s multiple comparisons test, p=0.0067 and 55% in CA1, p=0.00015; two-way ANOVA: no effect of brain region: F=3.0, p=0.094; no interaction between brain region and environment novelty: F=3.4, p=0.079). Not all of their studies used novel environments, however, and thus more differences remain to be accounted for.

**
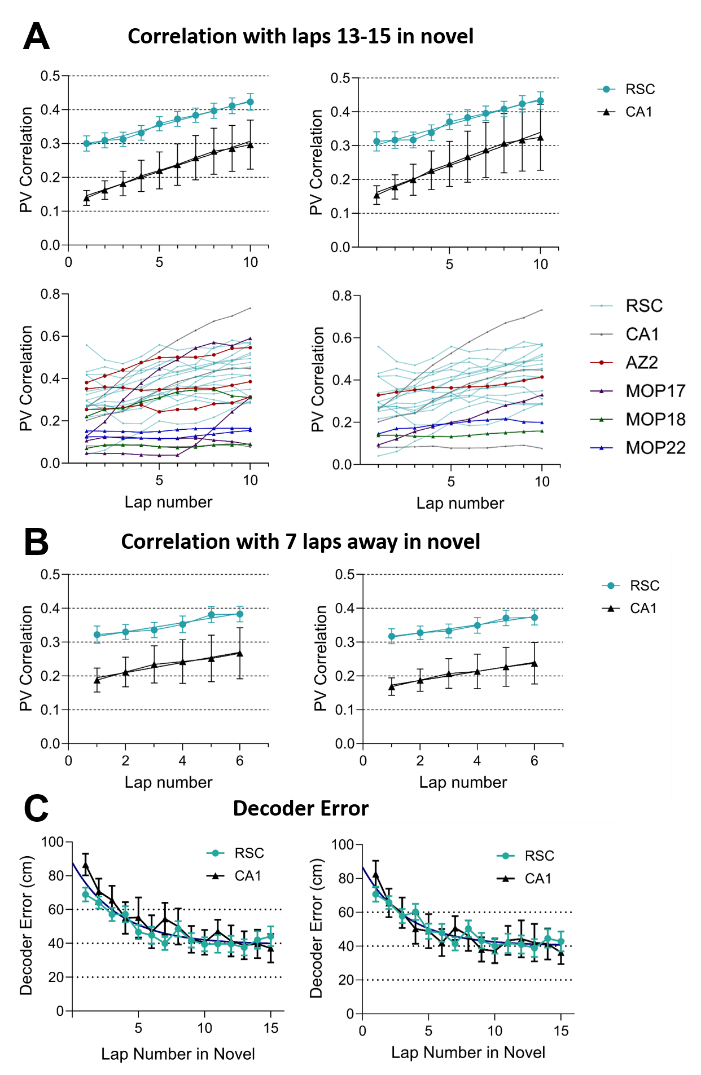
Figure S3: Datasets from the same mouse.**

With four mice, we collected multiple (2-3) datasets using different novel environments. For some analyses, (e.g. place field size), we averaged the results from those 2-3 datasets within a mouse before applying statistics across mice. However, in some analyses, we thought it was more appropriate to treat each novel environment as an independent sample, because there were large differences between environments within the same mouse. **A**. Correlations of population activity between early and later laps showed this. The top left plot shows the result displayed in Fig. 3D, using each session as an independent sample (RSC: n=18; CA1: n=10; linear regression: RSC: F(1,168)=37, p<0.0001; CA1: F(1,98)=9.4, p=0.0028). The top right shows the same data, now with the repeat datasets averaged within a mouse first and treated as a single sample (RSC: n=16; CA1: n=6; linear regression: RSC: F(1,158)=33, p<0.0001; CA1: F(1,58)=7.4, p=0.0086). The bottom left shows each dataset plotted individually, with datasets coming from the same mouse highlighted in the same color. This shows how different the response can be between datasets from the same mouse. For example, mouse MOP17 showed a spatial map growing in stability starting from lap 2 and plateauing at lap 8 in one novel environment, in a different environment, the spatial map didn’t start to become stable until after lap 6, and in a third environment there was no real stability within the first 15 laps. The bottom right panel shows that averaging these disparate responses generated a curve that was not representative of any of these individual environments, and thus we decided to treat them as independent samples. **B**. Same as the top panels in A, except now reproducing the analysis in figure 3E. Linear regression: left panel: RSC: F(1,100)=5.5, p=0.021; CA1: F(1,58)=1.5, p=0.22; Right panel: RSC: F(1,94)=6.0, p=0.016; CA1: F(1,34)=1.3, p=0.27. **C**. The decoder error results were also not different whether we used sessions as independent samples (RSC: n=17; linear regression: F=41, p<0.0001; CA1: n=10; F=28, p=0.0002) or averaged within mice (RSC: n=15; linear regression: F=41, p<0.0001; CA1: n=6, F=19, p<0.0001).

**Figure S4: Decoder error calculation.**

**
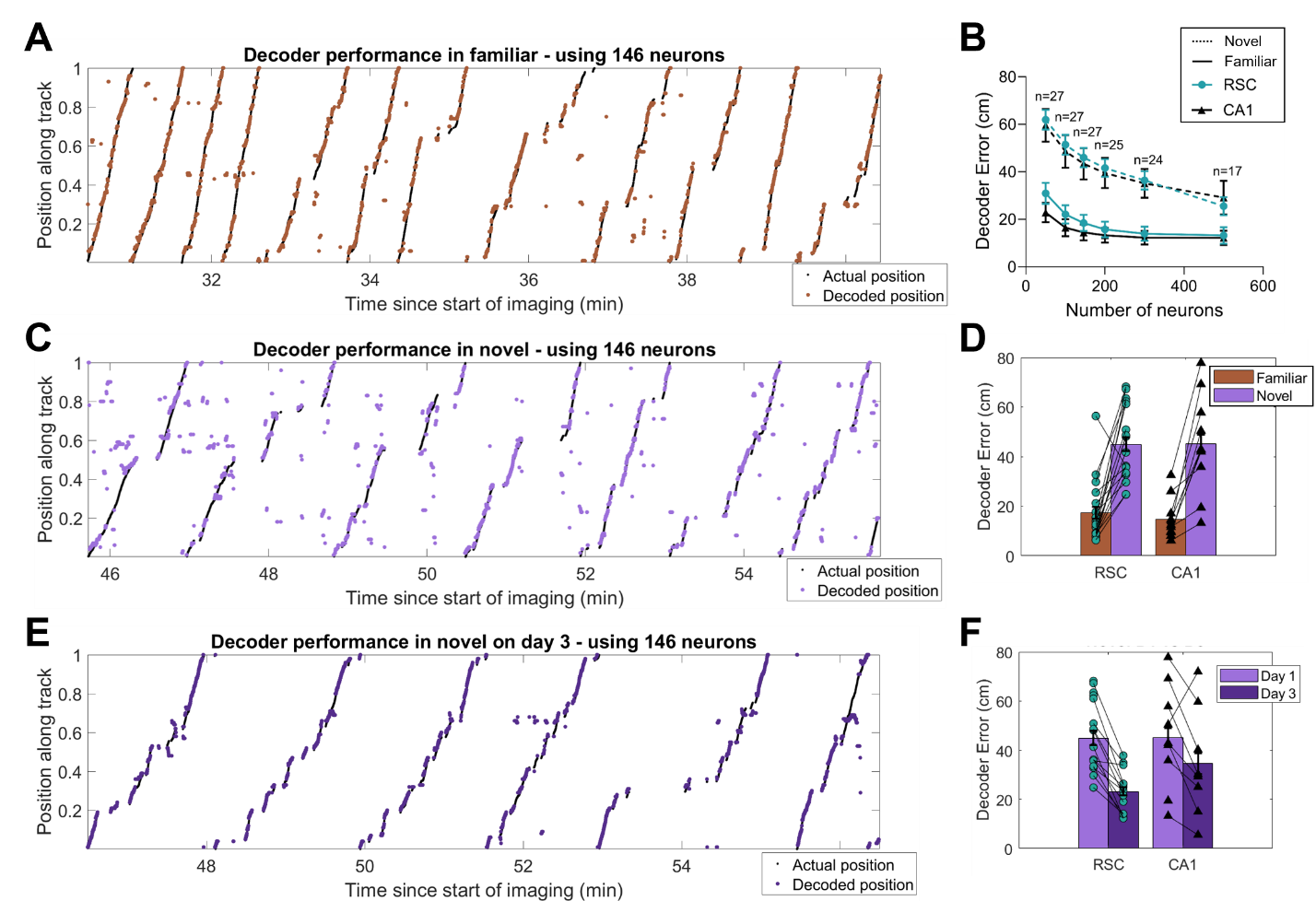
**

The Bayesian decoder was calculated using a random subsample of 146 neurons, 100 times, and then averaged across subsamples (see Methods). **A**. The decoder performance of a single subsample of 146 RSC neurons during a 10 min run period in a familiar environment. The actual position of the mouse is plotted in black, and the predicted position is marked by brown dots. Time bins during which the mouse was stopped (running at < 2cm/s) were excluded from analysis. The decoder error was calculated as the absolute value of the difference between the actual and the predicted position, averaged either within each lap, or across the whole session. **B**. We ran the decoder using 50, 100, 146, 200, 300 and 500 neurons. In the familiar environment, a plateau decoder performance was reached at 200 neurons, while in the novel environment it required more neurons. There were no differences in the curves between RSC and CA1 datasets (H0=one curve for all datasets: Novel: F(3,141)=0.11, p=0.95; Familiar: F(3,107)=1.2, p=0.31). 27/28 datasets included at least 146 neurons, and thus this number was used for all future analyses (one mouse with fewer recorded RSC neurons, DB26, was excluded from all decoder error analyses). **C**. Decoder performance during a 10 min run period upon first entry into a novel environment (same mouse as in A). **D**. Comparison of decoder errors in familiar and novel environments. Two-way repeated measures ANOVA: effect of novelty: F(1,25)=65, p<0.0001; effect of brain region: F(1,25)=0.073, p=0.79. **E**. Decoder performance during a 10 min run period on day 3 in the novel environment (same mouse and environment as in A). **F**. Comparison of decoder errors between day 1 and day 3 in the novel environments. Mixed effects analysis: effect of days: F(1,19)=34, p<0.0001; effect of brain region; F(1,25)=0.31, p=0.58.

**Figure S5: Running speed does not (entirely) explain calcium activity changes and between-lap correlation changes during novelty.**

**
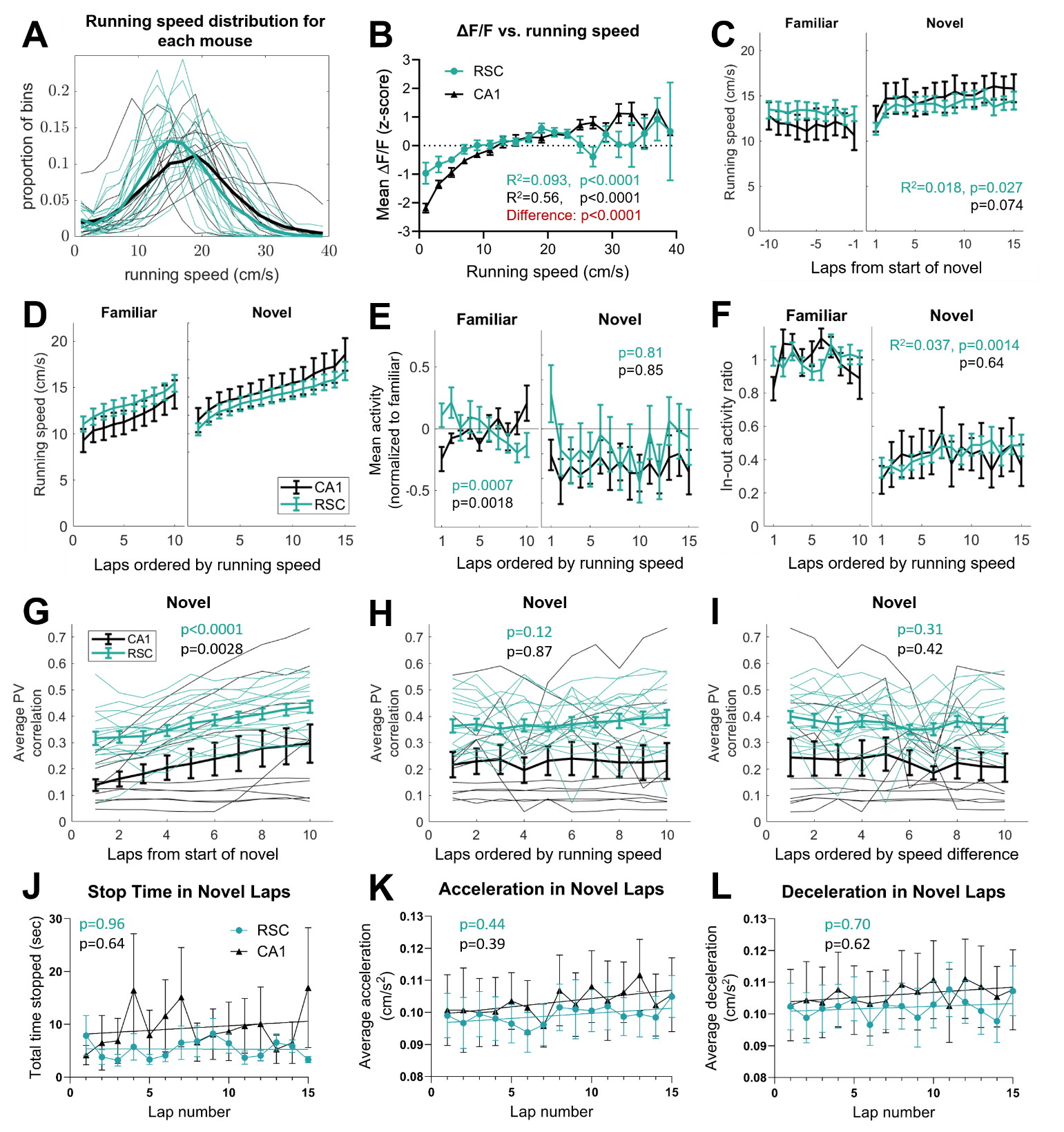
**

**A.** The running speed distribution for each mouse. Mice varied in their running speeds in our virtual reality setup, but most ran at peak speeds of 20-30 cm/s. **B**. To quantify the influence of running speed on neural activity, we found the running speed, and the mean activity across all cells in each lap (except lap 1 in familiar) and bin of the track. We binned both the running speed and the z-scored activity in 2 cm/s running speed intervals. Any intervals with less than 10 instances were removed for that mouse. Not all mice ran >25 cm/s, and thus there is more variability those intervals. There was a main effect of running speed on ∆F/F in both RSC and CA1. There was no effect of environment novelty, and so both Novel and Familiar environment laps were combined. A mixed effects analysis showed a significant effect of running speed, and a significant interaction with imaging area. Therefore, there was a difference between the running speed and neural activity relationship in RSC and CA1, but both regions individually also showed a main effect of speed. R^2^ and p values indicate the results of a linear regression for RSC (teal), and CA1 (black) datasets, and the difference in slopes between RSC and CA1 (red). **C**. The running speed in novel and familiar environments was only slightly different (faster in the novel environment), and the running speed increased slightly across laps in the novel environment (simple linear regression, all datasets combined, R^2^=0.019, p=0.0047). Turkey’s multiple comparisons test showed that lap 1 was significantly lower than most subsequent laps, and no other laps were different. **D**. To check if this relationship between running speed and lap number influenced any of our lap-based analyses, for each mouse, we sorted the laps based on running speed and then ran the same statistics tests as when laps were sorted in temporal order. **E**. Mean calcium activity across cells and each whole lap did not correlate with average lap speed (compare to Fig. 4C). **F**. In-out field activity ratio in RSC correlated slightly with mean lap running speed, but the same was not true for CA1 activity. The in-out activity ratio changed much more with temporally ordered lap number (Fig 4E). **G**. Population activity in each position bin across 3 continuous laps was correlated with activity in the same position bins during laps 13-15 (same as Fig 3D, except with individual datasets plotted as thin lines). **H**. The same continuous lap intervals were sorted based on the average running speed in each lap interval, now no longer showing a correlation across laps. **I**. Lap intervals were additionally sorted according the difference in running speeds with laps 13-15, again showing no relationship across sorted laps. All p values indicate the significance of a linear regression for RSC (teal) and CA1 (black) datasets. **J**. The total amount of time spent stopped (running at < 1 cm/s) was calculated for each lap. There was no effect of laps on stopping time in the first 15 laps in the novel environment. **K**. The mouse’s acceleration at each time point was calculated, and then all values >0 in each lap were averaged. The average acceleration did not change across laps. **L**. All acceleration values <0 in each lap were averaged and multiplied by -1. Deceleration did not change across laps. P values indicate the results of a linear regression for RSC (teal), and CA1 (black) datasets.

**Figure S6: Place field COM shift and field size measured using ∆F/F
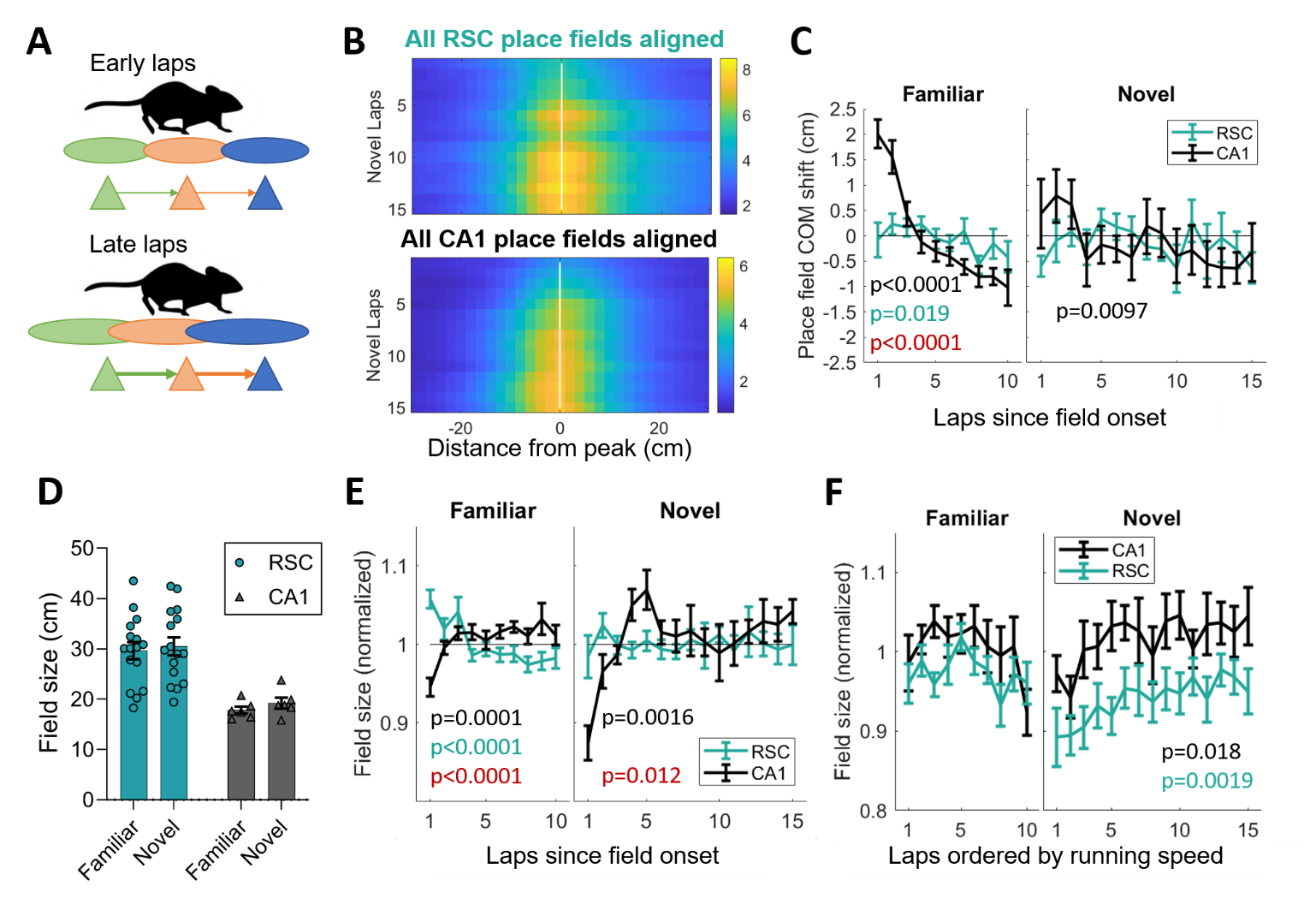
**

**A.** Schematic showing hypothetical place field expansion and center of mass shift from early to late laps, and the theorized mechanism. In early traversals of an environment, CA1 place cells (triangles) which express place fields at various locations along the track (ovals) are weakly connected. Across traversals along the same path, place cells with adjacent place fields strengthen their connections asymmetrically, causing post-synaptic cells to fire earlier, and expanding the place fields in the backwards direction. **B**. All RSC (top) and CA1 (bottom) place fields that were identified across mice were aligned to their peak location, and their ∆F/F was averaged for each lap. **C**. The COM was calculated for each place field in the familiar and novel environments by weighting each position bin within the boundaries of the field with the mean ∆F/F activity in that bin. Then the COM was calculated for each lap with above threshold activity, and subtracted from the mean COM for that field. The COM of all fields identified in a single session were averaged, before averaging across mice. CA1 cells showed a COM shift similar to previously published data. RSC fields showed a significantly smaller COM shift than CA1 in the familiar environment, and no significant shift in the novel environment. **D**. Field size was calculated for each place field by finding the peak ∆F/F activity within each lap, and counting the number of bins with activity at least half of that value. Averaged across laps, RSC place fields calculated in this way were significantly bigger than CA1 place fields. **E**. For each lap, the field size was normalized to the average across laps. Laps with mean activity below threshold were excluded from analysis, same as in C. CA1 fields increased in size across laps in both familiar and novel environments. RSC fields decreased in size across laps in the familiar environment, and showed no change in the novel environment. Error bars represent SEM. P-values indicate a significant correlation between the dependent variable and lap number for RSC (teal), or CA1 (black) datasets, or a significant difference between the slopes of RSC vs. CA1 datasets (red). **F.** For each mouse, laps were arranged based on running speed, instead of chronological order (same as in Fig S5 D-F). Then, normalized field sizes were averaged across mice, showing a gradual increase in field size with running speed in the novel environment in both RSC and CA1, but not the same abrupt increase observed between laps 1 to 5 in CA1 only.

Unlike in the main Figure 5, this analysis used fluorescence activity (∆F/F), instead of deconvolved calcium events to calculate field size and center of mass. It is unclear which measure is a more accurate representation of actual changes in place fields. The deconvolved calcium event-based measure is more similar to previously published data collected using electrophysiology, but it is more susceptible to noise, since it measures putative single events. The fluorescence-based method is influenced by the slow kinetics of the calcium indicator (which would cause fields to appear larger, especially when mice ran faster), and possibly the amount of activity. Running speed changes did not contribute to the observed increases in field sizes (see panel *F*), but perhaps these changes are a reflection of the fact that in-field neural activity increases across laps in the novel environment.

**Figure S7: Pupil movement changes associated with novelty.**


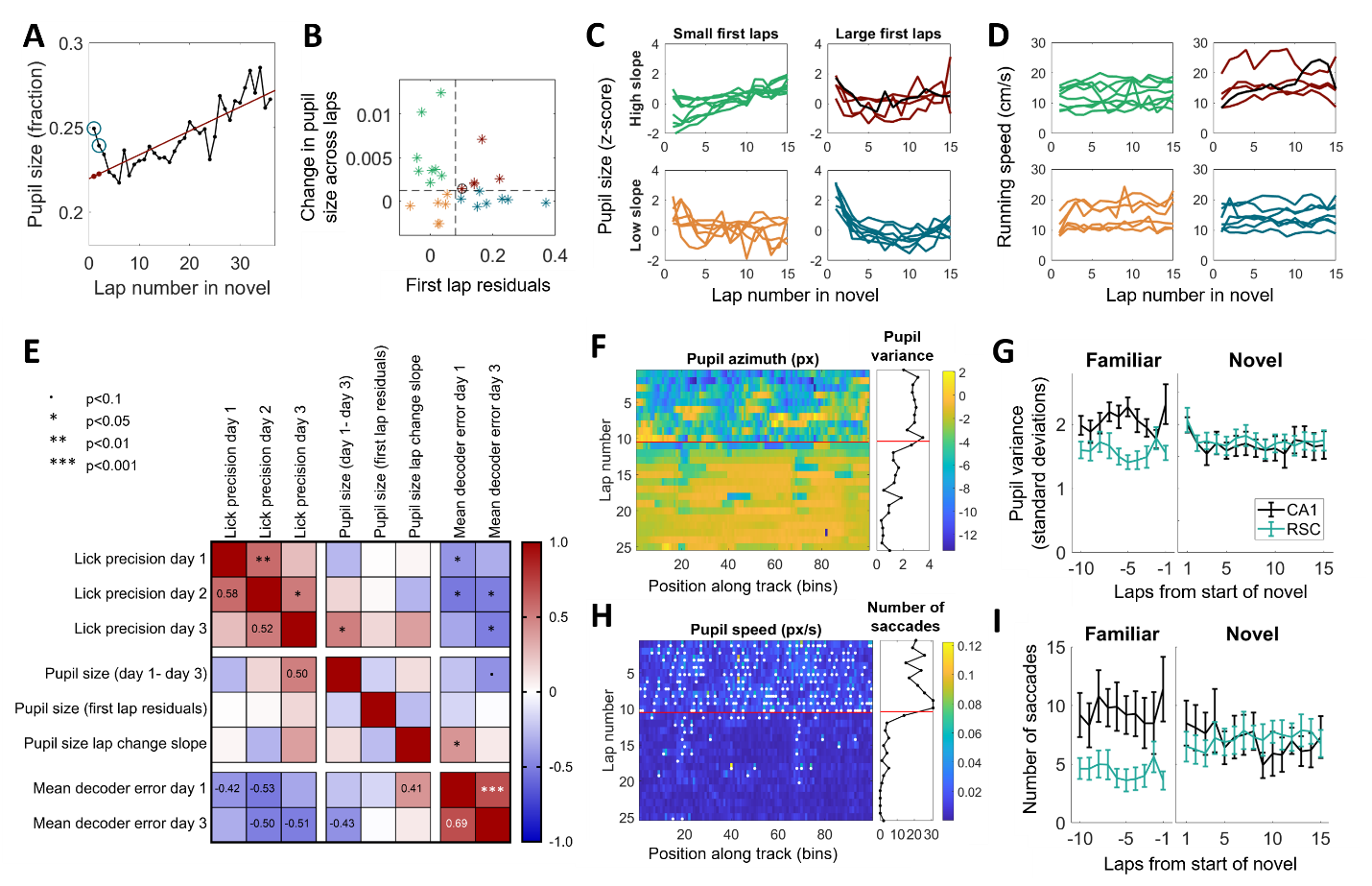


We analyzed several measures related to pupil size and movement. **A**. We identified two features of the evolution of pupil size across laps upon entry into a novel environment: There was a gradual increase in pupil size across laps, lasting the entire session, and, in the first couple of laps, pupil size was larger than in subsequent laps. These two features were computed for each dataset by measuring the slope of a best fit line (simple linear regression, red line); and the deviation of the first two laps (blue circles) from that best fit line (red dots; as a proportion of the size at the red dots). **B**. The change in pupil size across laps (best fit slope) and the deviation of the first two laps from this pattern (residuals) varied independently across datasets (linear regression: R^2^=-0.26, p=0.20). To illustrate the variability in pupil size changes, we classified each dataset into one of four categories (different colors), by splitting each variable roughly around the median value (dotted lines). The example dataset in A was circled in black. **C**. Pupil size across laps 1-15 in the novel environment was plotted for each dataset, split into the four categories identified in B. The example dataset from A was plotted in black. **D**. The increase in pupil size across laps was not due to running speed changes, as the running speed changes across laps did not differ between datasets with high versus low pupil size change across laps. **E**. We correlated the three pupil size measures (change between day 1 and day 3; the increase in size in laps 1 and 2, and the slope of pupil size change across all novel day 1 laps) with measures related to spatial encoding and memory (decoder error on days 1 and 3, and precision of licking at reward sites on days 1, 2 and 3). Significant correlations are marked by stars in the boxes above the diagonal, and their R^2^ value is indicated below the diagonal. The pupil size measures showed no correlation with each other, indicating that they represent independent features of the pupillary response (middle square). Interestingly, the fast pupil size response in laps 1 and 2 was not correlated with any other measures. The pupil size difference between day 1 and day 3 was significantly positively correlated with lick precision on day 3 (R^2^=0.50, p=0.049) and trended toward a significant negative correlation with decoder error on day 3 (R^2^=0.43, p=0.065). The slope of the change in pupil size across day 1 laps was positively correlated with decoder error on day 1 (R^2^=0.41, p=0.040). **F**. Pupil movement patterns changed noticeably between the familiar and novel environments in some datasets, as seen in this example. We measured the pupil azimuth variance (standard deviation) for each lap (right panel), to study if there was a systematic change in this behavior between novel and familiar. Familiar environment laps (top) are separated from novel environment (bottom) with a red line. **G**. Across datasets, there was no difference in pupil variance between familiar and novel environments (paired t-test, both tails: p=0.98), or between day 1 and day 3 (p=0.64; not shown). There was a small significant effect of laps on day 1 in the novel environment (Two-way ANOVA: effect of laps: F=2.1, p=0.049; effect of brain region: F=0.041; p=0.84), with lap 1 having a significantly higher variance than some of the subsequent laps (Tukey’s multiple comparisons test). **H**. We also measured pupil speed (left panel), and saccades (classified as pupil speed > 3 standard deviations above the mean; marked with white dots in left panel). As with pupil variance, some mice showed a noticeable change between familiar (above red line), and novel (below red line) laps in pupil speed and number of saccades per lap (right panel). **I**. However, across datasets, there was no consistent change in pupil speed (not shown) or number of saccades across environments (p=0.41), days (p=0.43; not shown), or laps (Two-way ANOVA: effect of laps: F=0.64, p=0.70). Datasets from RSC and CA1 are pooled throughout this figure.

**Supplementary videos SV1-SV3: Illustrations of the full VR environments.**

Each video shows a view of the left, front, and right tablets, which the mouse would see, along with a window containing the VR parameters which an experimenter would, during two passes through a single environment. Each video starts with blank tablets, which the mice see at the beginning of each session, and then the start of the first lap in the environment, followed by a second lap, and then a transition back into blank. The speed of movement is set to a constant 20 cm/s, except just prior to each reward site, where the video simulates the mouse slowing down, licking, receiving a reward, and then running again. Videos contain three environments: classroom, sunset, landscape.
